# Natural diversity-guided catalytic-core chimerism engineers a rapid and inhibitor-tolerant reverse transcriptase

**DOI:** 10.1186/s13036-026-00692-3

**Published:** 2026-05-07

**Authors:** Inês Fonseca Costa, Vânia Ondina Fernandes, Rita Silva Simões, Hélvio Simões, Virgínia Maria Rico Pires, Victor Diogo Alves, João Soeiro Teodoro, Pedro Bule, Carlos Mendes Godinho de Andrade Fontes

**Affiliations:** 1 NZYtech - Genes & Enzymes, Campus do Lumiar, Building J, Lisbon, 1649-038 Portugal; 2 CIISA - Centre for Interdisciplinary Research in Animal Health, Faculty of Veterinary Medicine, University of Lisbon, Lisbon, 1300-477 Portugal; 3 Associate Laboratory for Animal and Veterinary Sciences (AL4AnimalS), Lisbon, 1300-477 Portugal

**Keywords:** Reverse transcriptase, Protein engineering, Inhibitor resistant, Thermostable RT, M-MuLV RT, Protein chimera

## Abstract

**Background:**

Reverse transcriptases (RTs) are essential components of RNA-based molecular technologies, yet their practical performance is often constrained by trade-offs between catalytic speed, thermal stability, and tolerance to inhibitory substances. Conventional optimization via mutagenesis or directed evolution often improves individual traits but struggles to integrate multiple traits within a single enzyme. Here, we applied catalytic-core recombination, guided by natural sequence diversity and diagnostic performance criteria, to recover reverse transcriptase phenotypes that would be difficult to obtain by single-trait optimization alone.

**Results:**

Starting from 1,028 sequences, 24 representative RT variants were used to generate a library of chimeras by swapping a critical 405-residue polymerase domain into a validated M-MuLV scaffold. We identified chRT V18 as the lead candidate, demonstrating consistent activity across a broad temperature range (40–70 °C). In addition, chRT V18 enabled efficient and linear cDNA synthesis in as little as 1 min at elevated temperatures, while maintaining strong resistance to clinically relevant inhibitors. Beyond RT-qPCR, chRT V18 supported high-temperature RT-LAMP at 69 °C, a regime typically incompatible with conventional RTs, enabling improved specificity and rapid amplification. When formulated into one-step RT-qPCR master mixes, chRT V18 achieved low limits of detection and full concordance with IVD-approved assays, while reducing reverse transcription time by 90%, enabling shorter time-to-result.

**Conclusions:**

Natural diversity–guided catalytic-core chimerism enabled the development of a rapid, inhibitor-tolerant reverse transcriptase with an unusual combination of broad temperature compatibility, minute-scale reverse transcription, and resilience to clinically relevant inhibitors. Beyond the properties of chRT V18 itself, the results support modular recombination of evolutionarily optimized domains as a practical engineering strategy for integrating multiple performance traits in complex enzymes.

**Supplementary Information:**

The online version contains supplementary material available at 10.1186/s13036-026-00692-3.

## Highlights

This work uses catalytic-core chimerism as a practical engineering framework to recover reverse transcriptase phenotypes from evolutionary diversity.

Enables multi-objective optimization (temperature range, inhibitor tolerance, speed) without iterative mutational rounds.

Discovers chRT V18, a chimeric RT that supports efficient reverse transcription in a 1-minute step across 40–70 °C.

Validates cross-platform utility, enabling 69 °C RT-LAMP and shortened RT-qPCR time-to-result in inhibitor-rich matrices.

**Supplementary Information:**

The online version contains supplementary material available at 10.1186/s13036-026-00692-3.

## Graphical Abstract

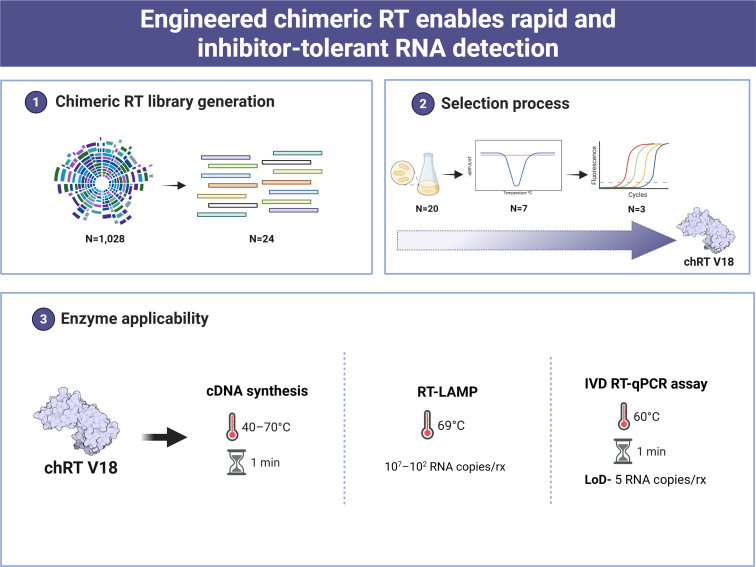

## Background

Reverse transcriptases (RTs) are indispensable tools for RNA-based molecular diagnostics, enabling the conversion of RNA into complementary DNA (cDNA) for downstream amplification and detection of infectious pathogens, identification of cancer RNA biomarkers, and profiling disease-related gene expression [1–3]. Despite substantial progress in reverse transcriptase engineering, important challenges remain in matching enzyme performance to the evolving demands of molecular diagnostics, particularly for rapid, inhibitor-resilient, and operationally flexible RNA detection workflows. These constraints prolong time-to-result and reduce robustness, particularly when testing complex or minimally processed clinical samples.

Conventional protocols using the Moloney Murine Leukemia Virus (M-MuLV) RT are typically carried out at lower temperatures (37–42 °C), which preserve RNA integrity but increase the risk of non-specific priming and unwanted amplification, particularly with complex or partially degraded samples [4, 5]. Higher reaction temperatures (50–70 °C) can alleviate these limitations by improving primer specificity and disrupting RNA secondary structures, thereby enabling more efficient cDNA synthesis, but most routinely used RTs do not function reliably across this temperature range.

To optimize cDNA synthesis, enzyme engineering has been widely used to improve RT thermostability, fidelity, and precision. While successful in specific contexts, these strategies often introduce trade-offs that compromise enzyme activity due to the unpredictability of mutation insertion. For example, although point mutations in residues involved in template-primer (T/P) binding improved substrate affinity, they also affected enzyme activity and stability relative to the wild-type (WT) RT variant [6]. As another example, in vitro evolution using compartmentalized ribosome display has yielded RT variants with enhanced thermostability and processivity through increased substrate-binding affinity, although in some cases these improvements were accompanied by a lower 50 °C-to-37 °C activity ratio, consistent with reduced thermal stability [7]. Although not yet fully explored, rational mutations designed to improve RT inhibitor tolerance are likely to impact other functional activities, such as specificity and sensitivity, compromising RT applicability in molecular diagnostics.

Over evolutionary timescales, retroviral RTs have evolved into efficient enzymes finely tuned to diverse hosts and environmental conditions. In contrast to protein engineering, evolutionary processes tend to select functional combinations that support enzyme fitness under physiological conditions [8]. Mining natural sequence diversity provides a powerful complement to conventional mutagenesis, enabling access to evolutionarily filtered structural solutions that may already integrate desirable functional traits [9]. Recent metagenomic and phylogenetic studies have revealed considerable sequence diversity among MuLV-related, gammaretroviral, and endogenous retroviral RTs, suggesting that unexplored variants may already encode desirable biochemical features with major impact in molecular diagnostics [10, 11].

Previous engineering studies have shown that reverse transcriptase robustness can be improved together with inhibitor tolerance, processivity, or fidelity, although these associations do not arise from thermostability alone [12, 13]. In parallel, directed-evolution studies have shown that reverse-transcription function can also be engineered in alternative polymerase scaffolds, including thermostable DNA polymerases with RT-PCR activity, polymerases combining DNA polymerase, RNA polymerase and reverse transcriptase activities in a single polypeptide, proofreading RTs such as RTX, and RTs evolved for diverse RNA and XNA templates [14–17]. In several cases, the underlying mechanisms involved tighter template-primer binding or enhanced nucleic-acid interactions, which improved resistance to inhibitory substances and supported more processive cDNA synthesis [12, 13]. In other studies, higher reaction temperatures improved processivity and fidelity by reducing pausing on structured RNA templates and decreasing extension of mismatched template-primers, rather than through a direct effect of thermostability itself [18, 19]. Enhanced thermostability and substrate-binding affinity may also promote greater sensitivity to low RNA inputs, enabling early disease detection and accurate quantification of rare transcripts [20]. Previous insights from thermostable DNA polymerases suggest that temperature stability is indeed linked to broader biochemical resilience, including resistance to proteolysis, pH fluctuations, and chemical denaturation [21]. Based on these considerations, we proposed that natural RTs with broad temperature adaptability would also exhibit enhanced resilience to inhibitors and biochemical stresses, making them well-suited for point-of-care and high-throughput diagnostics [22].

Here, we systematically explored the natural diversity of MuLV-related RTs and applied a catalytic-core chimerism strategy to integrate naturally evolved polymerase domains into a validated M-MuLV scaffold. The overall screening and prioritization workflow is summarized in Figure S1. This approach enabled the identification of chimeric RTs with improved performance characteristics, including the lead variant chRT V18, which supports rapid reverse transcription across a broad temperature range and shows strong potential for accelerated, robust RNA diagnostics.

This study expands the RT discovery landscape by showing that natural diversity can serve as a powerful complement to rational engineering approaches. Rather than presenting chimerism as a new concept, we use it here as a practical framework to identify reverse transcriptase phenotypes relevant to molecular diagnostics and next-generation RNA detection technologies.

## Materials and methods

### Mining natural MuLV RT diversity and phylogenetic analysis

To explore the natural biodiversity of RTs, a total of 1,028 protein sequences showing 70–100% sequence identity to M-MuLV RT (PubMed Protein Accession Number: AAC82568) were selected using protein BLAST (Basic Local Alignment Search Tool, NCBI, Bethesda, MD: https://blast.ncbi.nlm.nih.gov) [23]. The phylogenetic analysis was performed using the fully automatic workflow of NGPhylogeny.fr (Institut Pasteur, Paris, France: https://ngphylogeny.fr/workflows/oneclick/) [24]. The resulting phylogram was visualized and edited using TreeViewer [25]. Multiple sequence alignments were performed using the MAFFT (Multiple Alignment using Fast Fourier Transform) version 7.511.25 (Osaka University, Osaka, Japan: https://mafft.cbrc.jp/alignment/server/index.html) [26]. The obtained alignment of the natural RTs and controls was displayed using Aline and colored according to the ‘Gecos Flower’ coloring scheme [27].

### Gene synthesis, cloning, expression, and purification of RT variants

Synthetic genes encoding the selected RT domains were codon-optimized for expression in *Escherichia coli* (*E. coli*) using the ATGenium algorithm (NZYtech, Lisbon, Portugal), as described previously [28]. The synthesis of the selected 24 natural RT genes encompassed 405 amino acids, from Leu48-Leu452 of each natural RT. To facilitate cloning employing Gibson assembly, highly conserved sequences (aa 39–47 and aa 453–460) were added to the synthesized gene. The expression plasmids containing full-length M-MuLV WT RT and the engineered MuLV RT V1 were obtained from previous work. The full sequence of MuLV RT V1 is provided in a previous reference [22]. All chimeric constructs contained three-point mutations (D524G, E562Q, and D583N) to inactivate RNase H activity. The genes encoding RTX [17], native RT full-length (GenBank: QJT93250) and RT V18 homologue (GenBank: QJT93247) were synthesized similarly and cloned into the pHTP1 vector (NZYtech), whose sequence is publicly available on the company’s website. This vector was used for recombinant expression in *E. coli* and incorporates an N-terminal 6xHis tag for immobilized metal ion-affinity chromatography (IMAC) purification. All positive clones were fully sequenced in both directions to ensure 100% identity with the designed gene sequences.

The recombinant RT proteins were expressed in *E. coli* BL21 (DE3). Cells were cultured in 50 mL of NZY Auto-Induction LB medium (NZYtech) and incubated at 37 °C for 4 h. The temperature was then lowered to 15 °C, and cells were further incubated for 18 h. Cells were harvested by centrifugation and lysed using 7.5 mL of NZY Bacterial Cell Lysis Buffer (NZYtech) supplemented with 0.2 µg/mL of DNase I, and 100 µg/mL of Lysozyme. Recombinant proteins were purified by IMAC using His GraviTrap™ columns (Cytiva, Wilmington, DE) [29]. The purification protocol comprised a three-step washing protocol using 10 mL wash buffer A (50 mM Na_2_HPO_4_, pH 7.5, 1 M NaCl, 10 mM Imidazole) for the first 2 washes, and a final wash using 10 mL of wash buffer B (50 mM Na_2_HPO_4_, pH 7.5, 1 M NaCl, 22.5 mM Imidazole). Finally, proteins were eluted in 1 mL fractions using elution buffer (50 mM Na_2_HPO_4_, pH 7.5, 200 mM NaCl, 300 mM Imidazole). All purified proteins were quantified using NanoDrop™ One Microvolume UV-Vis Spectrophotometer (Thermo Fisher Scientific, Waltham, MA) and diluted to 0.7 mg/mL with the elution buffer. For long-term storage, proteins were diluted to 0.35 mg/mL with 50% (v/v) glycerol, supplemented with 1 mM DTT and 0.01% Triton X-100. Protein solubility, integrity, and purity were evaluated by SDS-PAGE. Unless otherwise stated, all assays were performed using the enzyme stock at a concentration of 0.35 mg/mL.

### Thermostability analysis and protein thermal shift assay

For thermostability analysis using SDS-PAGE, proteins were incubated at 4 °C, 45 °C, 50 °C, or 55 °C for 15 min. The incubation reactions were carried out in a total volume of 40 µL containing 1x Reaction Buffer for Reverse Transcriptases (NZYtech) and 1.05 µg of the respective RT. After the incubation, samples were cooled on ice for 15 min, followed by centrifugation at 12.500 rpm for 5 min, to pellet insoluble material. Then, 20 µL of the cleared supernatant was mixed with 5 µL of 5x SDS-PAGE Sample Loading Buffer (NZYtech), denatured at 95 °C for 10 min, and analyzed by 14% SDS-PAGE followed by Coomassie Blue staining. In addition, proteins were incubated at 45 °C for 0, 15, 30, 45, and 60 min and processed as before. Protein stability in these experiments was qualitatively evaluated by comparing the intensity and integrity of the soluble protein bands with those of the respective non-incubated controls. RT residual activity was also evaluated using one-step and two-step RT-qPCR. In both workflows, each RT was incubated at 50 °C, 52.5 °C, 55 °C, or 60 °C for 10 min before proceeding to the RT-qPCR setups as described in Sect.  2.4 and 2.5, respectively.

Melting temperatures (Tm) were determined using the Protein Thermal Shift™ Kit (Thermo Fisher Scientific), which monitors heat-induced protein unfolding through a fluorescent dye that binds exposed hydrophobic regions. The assay was performed in a CFX Opus 96 Real-Time PCR System (Bio-Rad). Each 20 µL reaction contained 5 µL of Protein Thermal Shift™ Buffer, 2.5 µL of Protein Thermal Shift™ Dye (8×), and 0.75 µg of the respective protein. Samples were heated from 25 °C to 95 °C at a ramp rate of 0.2 °C per 15 s, and fluorescence was recorded using the FRET channel. Tm was defined as the midpoint of the negative melt peak. All reactions were performed in triplicate.

### One-step RT-qPCR

To assess the reverse transcription activity, one-step quantitative reverse transcription polymerase chain reaction (RT-qPCR) reactions were performed using a customized master mix based on the commercially available Supreme NZY One-Step RT-PCR Master Mix 2x (NZYtech). Briefly, the RT included in the original formulation was replaced with 0.022 µg of the respective chimeric RT or control enzyme per reaction. Each 20 µL reaction contained 10 µL of the customized master mix, 0.4 µM of each primer, and 0.125 µM of TaqMan probe to amplify the mouse (*Mus musculus*) ribosomal protein L27 (*Rpl27*) or peptidylprolyl isomerase A (*Ppia*) housekeeping genes (expected amplicon sizes are given in Table S2). Mouse liver total RNA (Takara Bio Inc., Kusatsu, Shiga, Japan) was used as a template. Multiplex RT-qPCR assay was performed using COVID-19, Flu A/B, RSV Multiplex One-Step RT-qPCR Kit, IVD (MD0490, NZYtech). This assay targets six viral RNA sequences, SARS-CoV-2 (*RdRp* and *N* genes), influenza A, influenza B, respiratory syncytial virus (RSV) A and B, and a human ribonuclease P (RNase P) internal control. The reactions were incubated on the Applied Biosystems StepOnePlus™ Real-Time System (Applied Biosystems, Thermo Fisher Scientific, Waltham, MA) using the following protocol: the reverse transcription step was performed as indicated, followed by a 95 °C step for 2 min, then 40 cycles of 95 °C for 5 s and 60 °C for 30 s, with fluorescent signal measurement at the FAM™ channel. The reverse transcription efficiency was assessed using a standard curve with six serial dilutions of mouse liver total RNA. The qPCR efficiency was calculated by StepOne Software v2.3 (Applied Biosystems).

### Two-step RT-qPCR

To synthesize first-strand cDNA, a 10 µL RNA mixture containing 5 mM Oligo(dT)_18_ primer mix (NZYtech), 1 mM dNTPs (NZYtech), and mouse liver total RNA (Takara Bio Inc.) was incubated at 65 °C for 5 min, followed by 2 min on ice. A reaction mixture of 10 µL composed of 2x Reaction Buffer for Reverse Transcriptases (NZYtech), 40 U of NZY Ribonuclease Inhibitor (NZYtech), and 0.35 µg of the RT in test was added to the primed RNA mix, resulting in a final reaction volume of 20 µL. The final reaction mixture was incubated in T100 Thermal Cycler (Bio-Rad Laboratories, Inc., Hercules, CA) using the indicated protocol. Following cDNA synthesis, the enzyme was inactivated at 85 °C for 5 min. cDNA products were treated with RNase H at 37 °C for 10 min. The synthesized cDNA was quantified by real-time qPCR on the Applied Biosystems StepOnePlus™ Real-Time System (Applied Biosystems), targeting the mouse *Rpl*27 or *Ppia* genes (expected amplicon sizes are listed in Table S2). The qPCR reactions were carried out in a total volume of 20 µL containing 1x NZYSupreme qPCR Probe Master Mix (NZYtech), 4 µM of each primer, and 1.25 µM of TaqMan probe, using 2 µL of cDNA as the template. The following PCR protocol was used: 95 °C for 2 min, followed by 40 cycles of 95 °C for 10 s and 60 °C for 30 s, with fluorescence signals measured at the FAM™ channel. The reverse transcription efficiency in two-step RT-qPCR was assessed as described in Sect.  2.4.

### Resistance to PCR inhibitors assay

The inhibitor panel used in this study included the following contaminants: ethanol (PanReac AppliChem, Darmstadt, Germany), isopropanol (PanReac AppliChem), guanidine hydrochloride (Molekula Group, Darlington, UK), NZYol (RNA extraction reagent, NZYtech), sodium citrate dihydrate (Sigma-Aldrich, St. Louis, MO), animal blood containing potassium ethylenediaminetetraacetic acid (K-EDTA) (LAMPIRE Biological Laboratories, Thermo Fisher Scientific, Waltham, MA), and human plasma (Sigma-Aldrich). To evaluate RT resistance to PCR inhibitors, 5 µL of each contaminant (4× concentrated) were added to the reverse transcription reaction mixture before cDNA synthesis was initiated, and the reaction was then incubated at 50 °C for 10 min, as described in Sect.  2.5. In the control reaction, 5 µL of water was used instead. The inhibition of RT activity during cDNA synthesis was quantified by qPCR by calculating the difference between the C_T_ values of the inhibitor reaction and the control reaction (no inhibitor). This difference is referred to as ΔC_T_ (C_T_ Inhibitor-C_T_ No inhibitor). Each inhibitor was tested at two concentrations, and the concentration that produced the clearest differential response between M-MuLV WT RT and the engineered enzymes is shown in Fig. 3, whereas Figure S9 reports complementary data at a second inhibitor concentration.

### RT-LAMP assays

Real-time reverse transcription loop-mediated isothermal amplification (RT-LAMP) assays were performed to evaluate enzyme performance across varying input levels. A typical RT-LAMP reaction comprised of: 1x a customized master mix based on the commercially available Polaris^®^ Lyo RT-LAMP Master Mix (NZYtech) supplemented with 4 µg/mL of the reverse transcriptase (or the recommended level for the commercial enzymes for which enzyme concentrations are not provided) under test, 1 µL of template and water up to 25 µL. The reverse transcriptases tested were the following: chRT V18, GoScript™ Reverse Transcriptase (Promega Corp., Madison, WI), and RapiDxFire Thermostable Reverse Transcriptase (Lucigen, LGC Biosearch Technologies, Middleton, WI). The RT-LAMP master mix was benchmarked against WarmStart^®^ LAMP Kit (DNA & RNA) (New England Biolabs, Ipswich, MA) and Lyo-Ready™ RT-LAMP 1-Step Mix, 4x (Meridian Bioscience, Cincinnati, OH). The templates used in these assays were mouse liver total RNA (Takara Bio Inc.) to amplify the β-actin gene or the MS2 virus’s genome (Roche Diagnostics, Mannheim, Germany). All primer sequences are provided in Table S2. RT-LAMP reactions were conducted on the StepOnePlus™ Real-Time PCR System (Applied Biosystems) or Bio-Rad’s CFX Opus 96 (Bio-Rad Laboratories, Inc.), using the following protocol: isothermal incubation at 69 °C–65 °C for chRT V18, and at 65 °C for the commercial reverse transcriptases, with fluorescence acquisition every 30 s for 30 min, followed by an inactivation step at 95 °C for 3 min, and a final melt curve analysis with temperature increments of 0.5 °C at 10-second intervals over a range of 65 °C to 95 °C.

### One-step RT-qPCR master mix formulation and diagnostic validation

chRT V18 was formulated into one-step RT-qPCR master mixes in both liquid and lyophilized formats using Polaris^®^ (RT)-qPCR Buffer Y 2.5×, IVD (NZYtech), and Polaris^®^ Lyophilizable (RT)-qPCR Buffer Y 2.5×, IVD (NZYtech), respectively. Lyophilized mixes were reconstituted according to the manufacturer’s specifications. Performance was benchmarked against the SARS-CoV-2 One-Step RT-qPCR Kit II (NZYtech, MD0487), an IVD-approved assay, using the same primer-probe sets and cycling conditions, except for the reverse transcription step: 1 min at 60 °C for chRT V18 versus 10 min at 50 °C for the reference. Analytical sensitivity was assessed using LoD analysis, with quantified SARS-CoV-2 RNA spiked into nucleic acid extracts from clinically relevant matrices (e.g., whole blood, nasopharyngeal swabs, urine, cervical swabs). LoD values were expressed as copies µL⁻¹ and copies mL⁻¹ of sample input and defined as the lowest RNA concentration yielding ≥ 95% positive detection across replicates (*n* = 96). Assay precision and robustness were evaluated through repeated measurements across independent variables, including testing day, enzyme batch, operator, qPCR instrument, laboratory site, and clinical sample type. Both master mix formulations were assessed for repeatability, intra- and inter-assay variability, and coefficients of variation (CVs) of C_T_ values obtained across replicate runs and target concentrations, in accordance with standard diagnostic performance criteria. Clinical performance was evaluated using a panel of 32 previously characterized SARS-CoV-2 clinical samples (16 positive and 16 negative), tested in parallel using both the chRT V18-based master mixes and the IVD-approved reference assay. Concordance, sensitivity, specificity, and C_T_ value correlation relative to the reference assay were analyzed using consistent threshold settings across assays, with concordance defined as full agreement in qualitative results (positive/negative) between assays.

### Statistical analysis

Statistical analyses and graphical representations were performed using Microsoft Excel version 2512 (Microsoft Corp., Redmond, WA) and GraphPad Prism version 8.0.2 (GraphPad Software Inc., Boston, MA). Data are presented as mean ± SD from three technical replicates (*n* = 3), unless otherwise stated. Significant differences were evaluated by two-way ANOVA followed by Dunnett’s or Tukey’s multiple-comparisons post-hoc tests, as appropriate. Adjusted *P* values derived from these post-hoc comparisons were used to assign statistical significance, with *P* ≤ .05 considered significant.

## Results

### Mining natural MuLV reverse transcriptase diversity reveals extensive sequence variation within the polymerase core

**Fig. 1:**
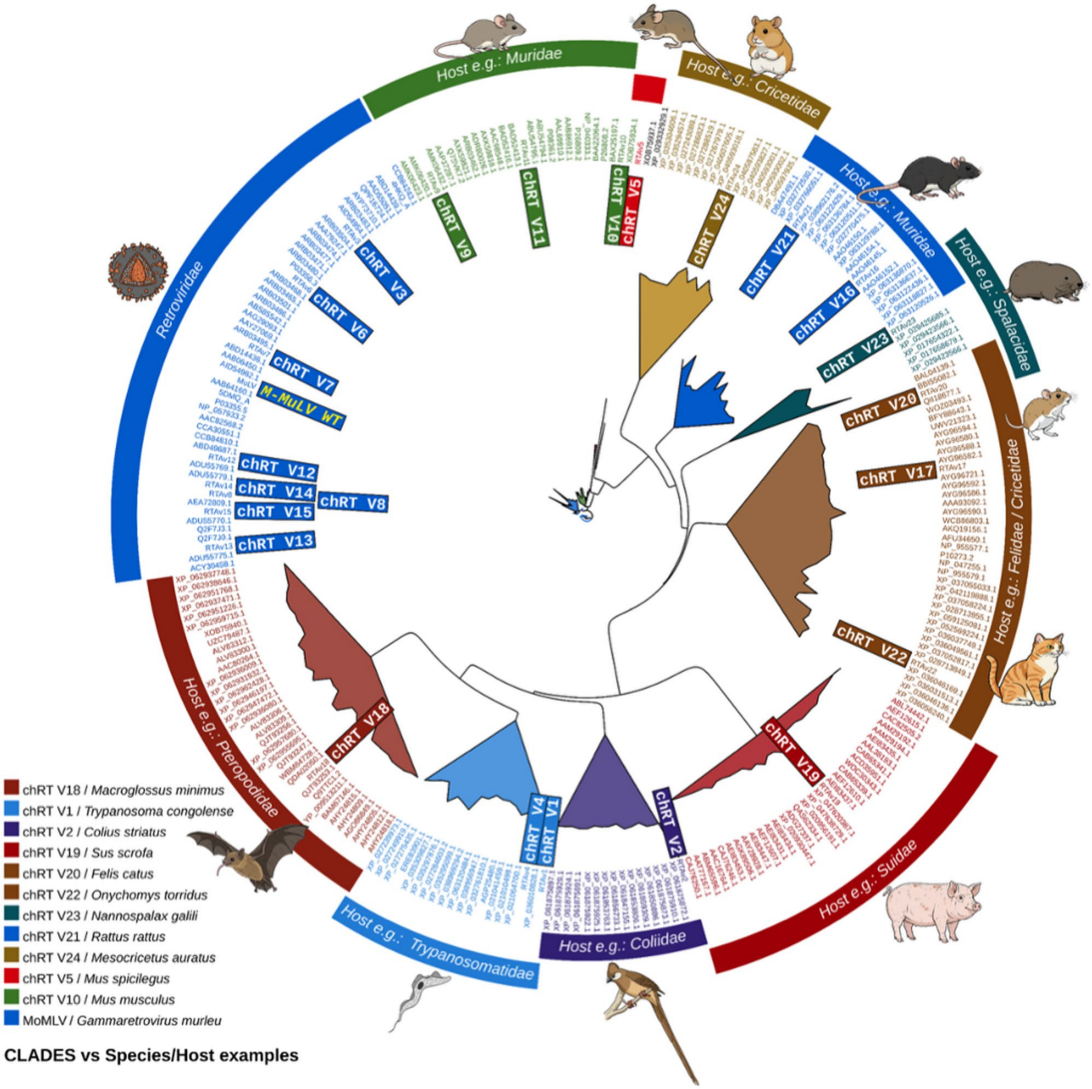
Phylogenetic tree of M-MuLV-related natural reverse transcriptase (RT) biodiversity. A phylogenetic tree representing the natural diversity of the initial 1,028-sequence dataset is shown, comprising the reduced, non-redundant dataset of 262 sequences. The 24 selected natural RT sequences are highlighted and colored by clade. The M-MuLV RT parental sequence is labeled yellow. Taxonomic diversity is illustrated by a representative species or host species for each clade

Viral RTs have undergone extensive evolutionary diversification [30, 31]. To systematically explore the natural diversity of RTs, we collected and analyzed a dataset comprising 1,028 RT sequences that share > 70% sequence identity with M-MuLV RT. For comparative analysis, we focused on a 405-residue segment (Leu48-Leu452) encompassing the core RNA-dependent DNA polymerase domain, which has been previously implicated in modulating enzymatic efficiency, fidelity, and thermal stability [32, 33]. To minimize redundancy while preserving sequence diversity, we constructed an initial unrooted phylogenetic tree from all 1,028 RT variants. We then derived a reduced dataset of 262 sequences by removing highly homologous entries (> 99% identity), while retaining the full spectrum of sequence variation present in the original dataset (Fig. 1). Phylogenetic analysis based on multiple sequence alignments guided the selection of 24 RT variants that represent the natural diversity of MuLV-related RTs (Table S1). As shown in Fig. 1, some selected sequences (e.g., chRT V3, V6, V7, V8, V12-V15) are closely related to M-MuLV, whereas others (e.g., chRT V1, V4, V18) display substantial sequence divergence. A multiple sequence alignment of the 24 selected RTs against M-MuLV WT confirmed diversity within the 405-residue region (Figure S2). Across the Leu48-Leu452 region, the selected variants shared a mean amino-acid identity of 87.9% relative to M-MuLV WT (range 69.4–98.3%), while mean pairwise identity among the variants was 81.3% (range 67.4–98.5%). More than half of the positions were polymorphic (218/405, 53.8%), and 70/405 positions (17.3%) exhibited four or more amino acid states, consistent with a broad sampling of natural sequence diversity without length variation across this domain. Several selected variants contain substitutions previously associated with altered enzymatic properties, such as thermostability or primer-template interactions, while others harbor sequence motifs that have not been functionally characterized [6, 7, 22, 34–36]. Both conserved and variant-specific substitutions were observed across the dataset, providing a diverse sequence basis for subsequent functional evaluation [37].

### Construction and expression of a chimeric RT library based on natural polymerase-core diversity

From the 24 selected MuLV RT variants, we generated a corresponding library of chimeric enzymes. Each chimera was constructed by replacing the 405-residue region (Leu48-Leu452) of wild-type M-MuLV RT with the corresponding sequence from one selected variant, yielding 24 chimeric enzymes, hereafter termed chRT V1-V24. The M-MuLV RT backbone included a C-terminal inactivated RNase H domain through three-point mutations (D524G, E562Q, D583N). Gibson assembly enabled seamless cloning, and all synthetic genes were codon-optimized for expression in *E. coli*. The 24 RT chimeras were expressed in *E. coli* BL21 (DE3) and purified by IMAC [29]. Protein purity and integrity were assessed by SDS-PAGE, as illustrated in Figure S3. While most chimeras yielded soluble enzymes, chRT V1, V2, V6, and V7 exhibited poor solubility or failed to express [38]. Yields for the remaining 20 soluble RTs are reported in Table S3. These differences in expression and solubility indicate that sequence variation within the exchanged region influences these properties in a heterologous expression system, as also observed previously for engineered M-MuLV RT variants [39]. As functional controls, three recombinant RT enzymes were produced and purified as controls: (i) full-length M-MuLV WT RT; (ii) M-MuLV V1, previously characterized for enhanced thermostability and molecular diagnostic potential and containing an inactivated RNase H domain [22]; and (iii) RTX, a thermostable KOD DNA polymerase derivative engineered for RT activity [17]. Soluble RT chimeras, along with the three control enzymes, were tested for activity in a cDNA synthesis reaction at 50 °C for 30 min.

To identify chRT variants with enhanced thermal robustness, the 20 soluble chimeric enzymes, together with the three control RTs, were incubated at 4 °C, 45 °C, 50 °C, and 55 °C for 15 min, followed by SDS-PAGE analysis of the soluble fraction to assess protein integrity. As shown in Figure S4 and summarized in Table S4, stability varied substantially across the library. Several variants, including chRT V8, V11, V13, V15, V17, and V19-V24, showed visible degradation or aggregation already at 45 °C. In contrast, a smaller subset retained intact protein bands at 45–50 °C, with RTX remaining stable up to 55 °C. Based on this initial screen, seven chimeric RTs, chRT V3, V5, V9, V10, V12, V14, and V18, were selected as the most thermostable candidates for further analysis.

A time-course degradation assay at 45 °C was then performed with these seven candidates, again using SDS-PAGE to monitor soluble protein integrity over time (Figure S5 and Table S5). Most variants remained stable for at least 30 min, and chRT V3, V9, and V12, together with the control enzymes M-MuLV WT RT and RTX, retained visible protein bands after 60 min of incubation. To complement these qualitative assays with a quantitative biophysical measure, the melting temperatures of the seven selected chimeras were determined by protein thermal shift assay in parallel with M-MuLV WT RT (Figure S6). Six of the seven chimeras displayed melting temperatures above that of the WT scaffold, supporting the conclusion that catalytic-core replacement generally increased thermal stability. chRT V14 was the main exception, showing a melting temperature similar to that of M-MuLV WT RT. Because thermostability in the present workflow was evaluated using multiple orthogonal criteria, including heat-incubation band integrity and time-dependent degradation behavior, chRT V14 was retained for downstream functional analysis despite its WT-like Tm. Collectively, these data support the prioritization of chRT V3, V5, V9, V10, V12, V14, and V18 for RT-qPCR-based functional characterization.

### Functional screening identifies chimeric RTs with robust activity across an expanded temperature range

**Fig. 2:**
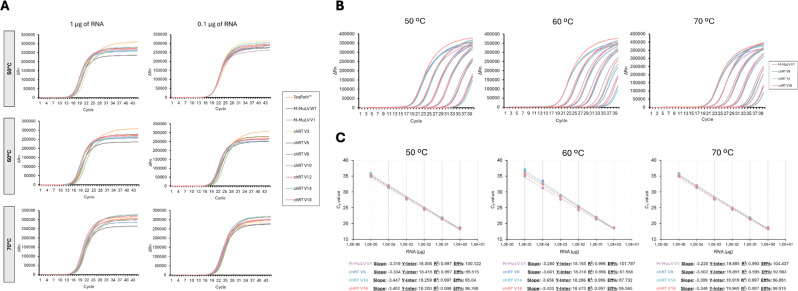
Reverse transcription performance of thermostable RT variants at different temperatures. (**A**) One-step RT-qPCR assays were performed using either 1 µg (left panels) or 0.1 µg (right panels) of mouse liver total RNA as template for cDNA synthesis at 50 °C, 60 °C, or 70 °C for 10 min, followed by amplification of the mouse *Ppia* housekeeping gene. Amplification curves are shown for the nine selected thermostable chimeric RT variants (chRT V3-V5, V9, V10, V12, V14, V16, and V18), together with M-MuLV WT, M-MuLV V1, and the commercial TaqPath™ 1-Step RT-qPCR Master Mix. (**B**) Two-step RT-qPCR amplification plots obtained using a 10-fold serial dilution of mouse liver total RNA, corresponding to approximately 5 × 10^6^ to 50 target copies per reaction. Reverse transcription was carried out at 50 °C, 60 °C, or 70 °C for 10 min using M-MuLV V1, chRT V9, chRT V14, or chRT V18, followed by qPCR detection of the mouse Ppia transcript. (**C**) C_T_-versus-input standard curves derived from the data shown in panel B. Linear regression parameters, coefficients of determination (R²), and amplification efficiencies are indicated for each enzyme at 50 °C, 60 °C, and 70 °C

Based on thermostability profiling, the functional performance of the seven selected RT variants was evaluated in one-step RT-qPCR assays. cDNA synthesis was performed at 50 °C, 60 °C, and 70 °C for 10 min, using either 1 µg or 0.1 µg mouse liver total RNA as template (corresponding to approximately 5 × 10^6^ and 5 × 10^5^ RNA copies per reaction, respectively). The seven variants were tested alongside M-MuLV WT and M-MuLV V1. RTX was excluded from subsequent one-step RT-qPCR comparisons because preliminary benchmarking in the assay configuration used in this study showed substantially delayed amplification and reduced fluorescence relative to the other reference enzymes across the temperatures and RNA inputs tested. TaqPath™ 1-Step RT-qPCR Master Mix (Applied Biosystems) was included as a commercial reference. As shown in Figs. 2A and S7A, all variants displayed a capacity to synthesize cDNA at 50 °C, 60 °C, and 70 °C, although differences in performance became apparent at elevated temperatures. To quantify temperature robustness, ΔC_T_ (70 °C–50 °C) was calculated for the two template quantities tested (Figure S7B). chRT V9, V14, and V18 consistently showed lower mean ΔC_T_ values than M-MuLV WT RT across both RNA input levels (Figure S7), suggesting better retention of reverse transcription performance at higher temperatures under the conditions tested.

To further evaluate thermal tolerance, chRT V9, V14, and V18 were pre-incubated for 10 min at increasing temperatures (50–60 °C) before one-step and two-step RT-qPCR assays. A non-incubated control was included for each enzyme. Loss of activity following pre-incubation was quantified as ΔC_T_ relative to the non-incubated control. The data, presented in Figure S8, revealed that all three enzymes retain their activity after incubation at 50 °C but became progressively less efficient as the incubation temperature increased, with ΔC_T_ values exceeding 5 cycles at 60 °C. In the one-step format, chRT V9 and V18 maintained stability up to 55 °C, whereas chRT V14 showed decreased performance at 55 °C and above. In contrast, all three variants showed instability at 55 °C or above when evaluated in the two-step RT-qPCR format under the conditions tested.

Sensitivity and linearity were subsequently evaluated for chRT V9, chRT V14, and chRT V18 using a two-step RT-qPCR assay across a 10-fold serial dilution of mouse liver total RNA, down to approximately 5 target copies per qPCR reaction. To assess performance across a broad temperature range, cDNA synthesis was performed between 50 °C and 70 °C, and the well-characterized thermostable M-MuLV V1 enzyme served as a control. The data presented in Fig. 2B and C revealed that the three enzymes and the control maintained linear amplification across the input temperatures tested at 50 °C, 60 °C, and 70 °C. Notably, chRT V18 showed the highest cDNA synthesis efficiency at 60 °C and 70 °C across the conditions tested, indicating increased robustness at elevated reaction temperatures. Overall, these results indicate that chRT V9, V14, and V18 remain active across a wide temperature range, with chRT V18 exhibiting the most consistent performance at higher temperatures.

### Chimeric RTs display resistance to clinically relevant PCR inhibitors

For diagnostic use, RTs must function reliably in crude or minimally processed samples, where inhibitory substances are common [18, 40]. To evaluate robustness under inhibitory conditions relevant to diagnostic workflows, chRT V9, V14, and V18 were tested against a panel of clinically relevant PCR inhibitors at sample-relevant concentrations (Table S6). In this assay, inhibitors were present during the reverse transcription step, and their effect was quantified indirectly from the resulting cDNA yield by qPCR. Reverse transcription was performed at 50 °C for 10 min for inhibitor-tolerance comparisons. The engineered M-MuLV V1 and the native M-MuLV WT RT were included as an inhibitor-tolerant and sensitive controls under these conditions [12]. Inhibitor resistance was quantified as ΔC_T_ between reactions performed in the presence and absence of each inhibitor (control reaction). A ΔC_T_ value ≤ 1.0 was used as a threshold for negligible inhibition, corresponding to a less than 2-fold reduction in amplification efficiency.

**Fig. 3:**
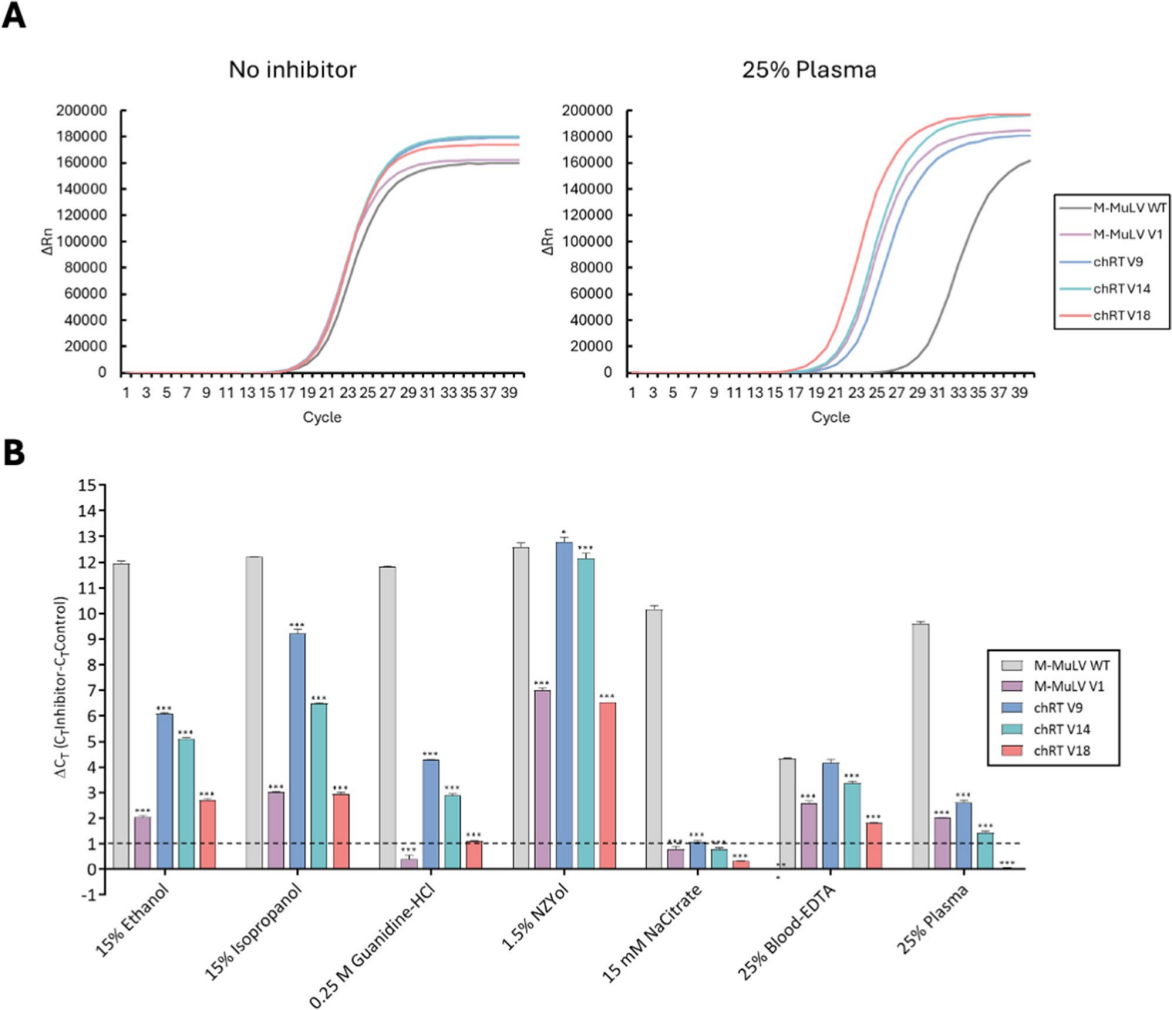
Inhibitor tolerance of WT, engineered, and chimeric RTs under the most discriminatory inhibitor conditions. (**A**) Representative amplification plots illustrating two-step RT-qPCR performance in the absence and presence of 25% (v/v) human plasma. (**B**) ΔC_T_ values for M-MuLV WT RT, M-MuLV V1, chRT V9, chRT V14, and chRT V18 in the presence of clinically relevant PCR inhibitors. Reverse transcription was performed at 50 °C for 10 min using 1 µg of mouse liver total RNA as template. Inhibition was quantified as ΔC_T_ (Inhibitor - No inhibitor). The dashed line at ΔC_T_ = 1.0 indicates the threshold for negligible inhibition. For each inhibitor, the concentration shown was selected as the most informative condition for resolving differences between M-MuLV WT RT and the engineered enzymes (see Figure S9 for alternate concentrations). Asterisk (*) and (***) indicate a statistically significant difference of *p* < .05 and *p* < .0001, respectively, compared against the M-MuLV V1 RT control, as evaluated by two-way ANOVA testing with Dunnett’s *post-hoc* analysis

As shown in Fig. 3, M-MuLV WT RT exhibited the greatest susceptibility under the selected inhibitor conditions, with pronounced ΔC_T_ increases in the presence of 15% (v/v) ethanol, 15% (v/v) isopropanol, 0.25 M guanidine hydrochloride, 1.5% (v/v) NZYol, 15 mM sodium citrate, and 25% (v/v) plasma. At the lower blood-EDTA and plasma concentrations, 15% (v/v), the inhibitory effect on M-MuLV WT RT was markedly reduced (Figure S9). In contrast, M-MuLV V1 retained strong overall inhibitor tolerance (Fig. 3), with ΔC_T_ values remaining close to or below the inhibition threshold for most conditions tested. The three chimeric enzymes also showed clear improvements over M-MuLV WT RT, except in the presence of 1.5% (v/v) NZYol, indicating that catalytic-core replacement conferred measurable resistance to several diagnostically relevant contaminants. Among the chimeras, chRT V18 generally displayed the most favorable profile, with ΔC_T_ values consistently lower than those of M-MuLV WT RT and, for several inhibitors, close to or even lower than those observed for M-MuLV V1. chRT V9 and chRT V14 also showed improved inhibitor tolerance relative to WT, although their performance was more variable across inhibitor identity and concentration. At the higher concentrations of ethanol, isopropanol, guanidine hydrochloride, and NZYol (Figure S9), none of the engineered enzymes showed a clear advantage over M-MuLV WT RT. Together, these results indicate that the selected chimeric RTs, and particularly chRT V18, preserve reverse transcription activity under several chemically and biologically relevant inhibitory conditions, whereas M-MuLV WT RT remains more sensitive under the same assay framework.

### chRT V18 enables efficient and fast cDNA synthesis across a broad temperature range

**Fig. 4:**
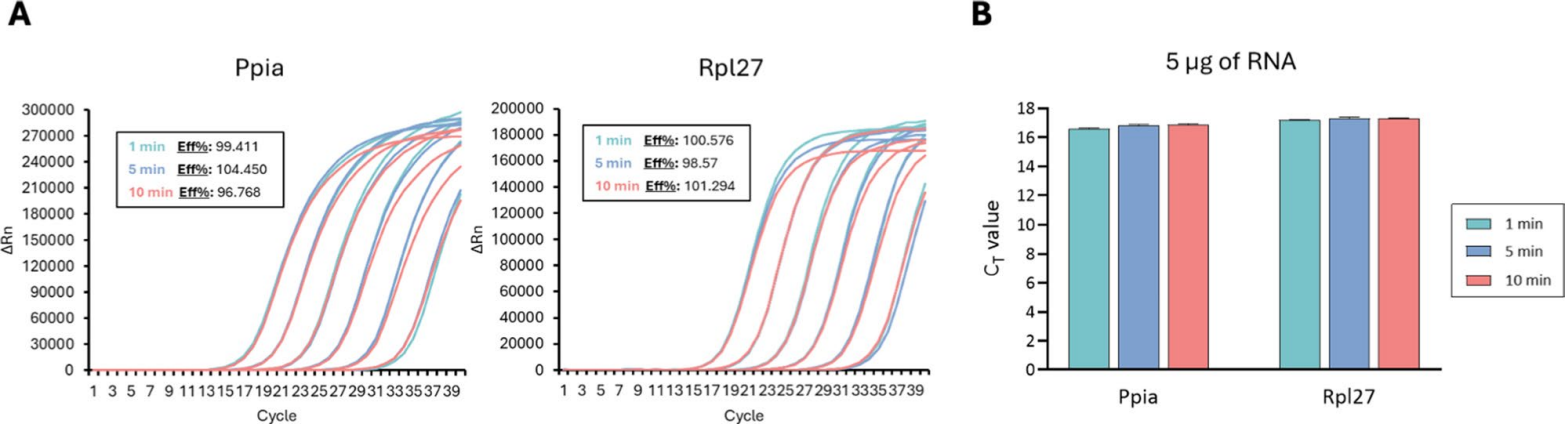
Rapid cDNA synthesis by chRT V18 at 60 °C. cDNA synthesis was performed using chRT V18 (glycerol-preserved format) at 60 °C in a two-step reverse transcription protocol with incubation times of 1, 5, or 10 min. Mouse liver total RNA was used as input over a six-point dilution series ranging from 5 µg to 5 pg, with oligo(dT) priming to enable reverse transcription of the full mRNA population. (**A**) RT-qPCR amplification curves for the mouse Ppia and Rpl27 transcripts following cDNA synthesis at the three reaction times, illustrating efficient cDNA generation across the full RNA input range. (**B**) C_T_ values obtained from reactions containing 5 µg of total RNA, showing comparable amplification across all reverse transcription times. No significant differences between compared experimental groups (*n* = 3) were observed, as evaluated by two-way ANOVA, followed by *post hoc* Tukey’s multiple comparisons tests

Based on the combined thermostability and inhibitor-resistance analyses, chRT V18 emerged as the most robust enzyme among the chimeric RTs evaluated. The speed of cDNA synthesis by this variant was therefore examined in greater detail under conditions relevant to diagnostic workflows. Initially, cDNA synthesis was performed at 60 °C using a two-step reverse transcription protocol with reaction times of 1, 5, or 10 min. Mouse liver total RNA was used as input at six starting quantities, ranging from 5 µg to five successive 10-fold dilutions. cDNA yield and efficiency were assessed using two endogenous mouse transcripts, Ppia and Rpl27. As shown in Fig. 4A and S10, efficient cDNA synthesis was observed across the entire dilution series for all three incubation times. Comparison of C_T_ values obtained from 5 µg RNA (Fig. 4B) showed no significant differences between 1-, 5-, and 10-minute reaction times, indicating that 1 min was sufficient to convert even high RNA quantities into amplifiable cDNA under these conditions.

**Fig. 5:**
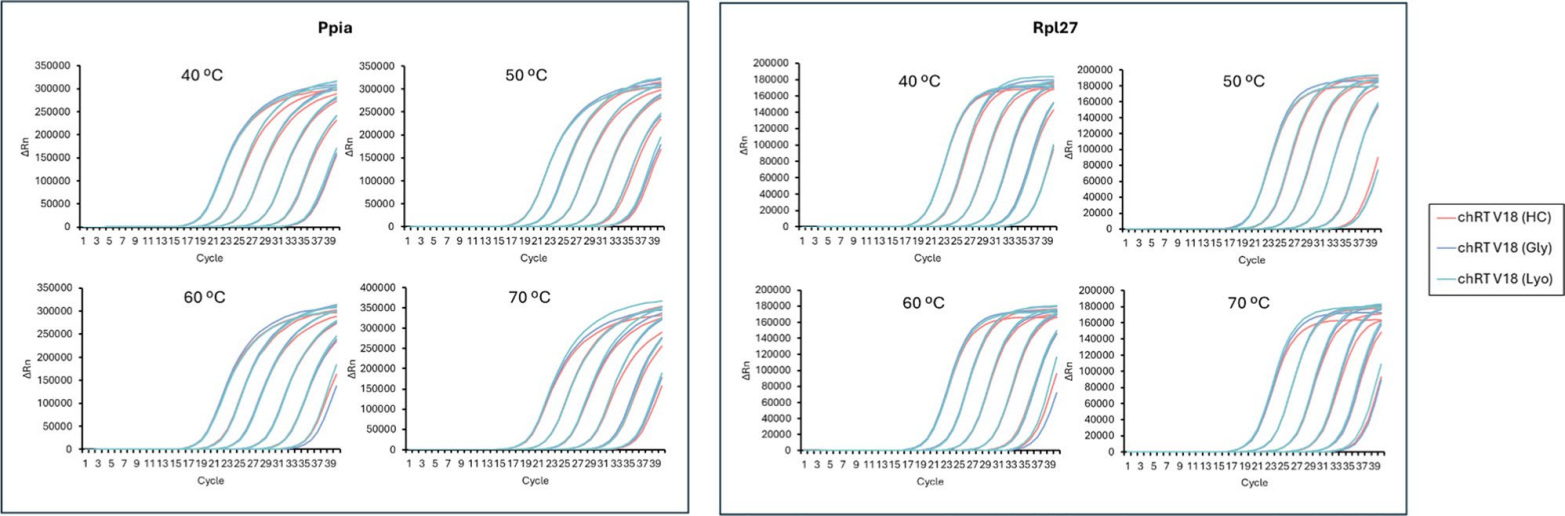
One-minute cDNA synthesis by chRT V18 across a broad temperature range and enzyme formulations. The ability of chRT V18 to support rapid cDNA synthesis across a wide temperature range was evaluated using a 1-minute reverse transcription step performed at 40 °C, 50 °C, 60 °C, or 70 °C. Mouse liver total RNA was used as template over a six-point dilution series (1 µg to 10 pg), with oligo(dT) priming. chRT V18 was tested in three formats: glycerol-free high-concentration (chRT V18 HC), glycerol-preserved enzyme at standard concentration (chRT V18 Gly), and lyophilized formulation (chRT V18 Lyo). RT-qPCR amplification curves are shown for the mouse Ppia (left panel) and Rpl27 (right panel) transcripts

To determine whether this rapid reaction capability was maintained across a broad temperature range, cDNA synthesis was performed for 1 min at temperatures ranging from 40 °C to 70 °C in a two-step experiment, followed by qPCR quantification. The inclusion of 40 °C in these assays was intended to assess whether chRT V18 retained robust and rapid activity across a broad operational temperature range, since flexibility between moderate and elevated reverse transcription temperatures is advantageous for different molecular diagnostic workflows. These experiments were conducted using chRT V18 in three formats: glycerol-free at high-concentration, glycerol-preserved at standard concentration, as well as lyophilized. The data, presented in Fig. 5 and S11), revealed that efficient, linear amplification was maintained across the full temperature range for both target genes, with comparable performance observed among the three enzyme formats. Taken together, the data demonstrate that chRT V18 retains efficient cDNA synthesis under a wide range of temperatures, reaction times, inhibitory conditions, and formulation states. This level of operational versatility is well aligned with the demands of modern molecular diagnostics, which frequently rely on short reaction times and flexible testing.

### The diagnostic performance of chRT V18 is encoded within its catalytic core and enhanced by scaffold context

To determine whether the functional properties identified for chRT V18 originate from the transferred catalytic core rather than from the M-MuLV backbone itself, the activity of chRT V18 was compared with that of multiple related enzyme structural analogs. These included a truncated RT V18 variant lacking the C-terminal RNase H domain (RT V18 TR), the full-length native RT V18 enzyme (RT V18 FL), and a closely related natural homologue of RT V18 FL (RT V18 homologue) sharing 94.9% amino acid identity. Wild-type M-MuLV RT in full-length (M-MuLV WT FL) and truncated (M-MuLV WT TR) forms were included as reference. All six recombinant enzymes were expressed in *E. coli* and purified to comparable purity (Figure S12).

**Fig. 6:**
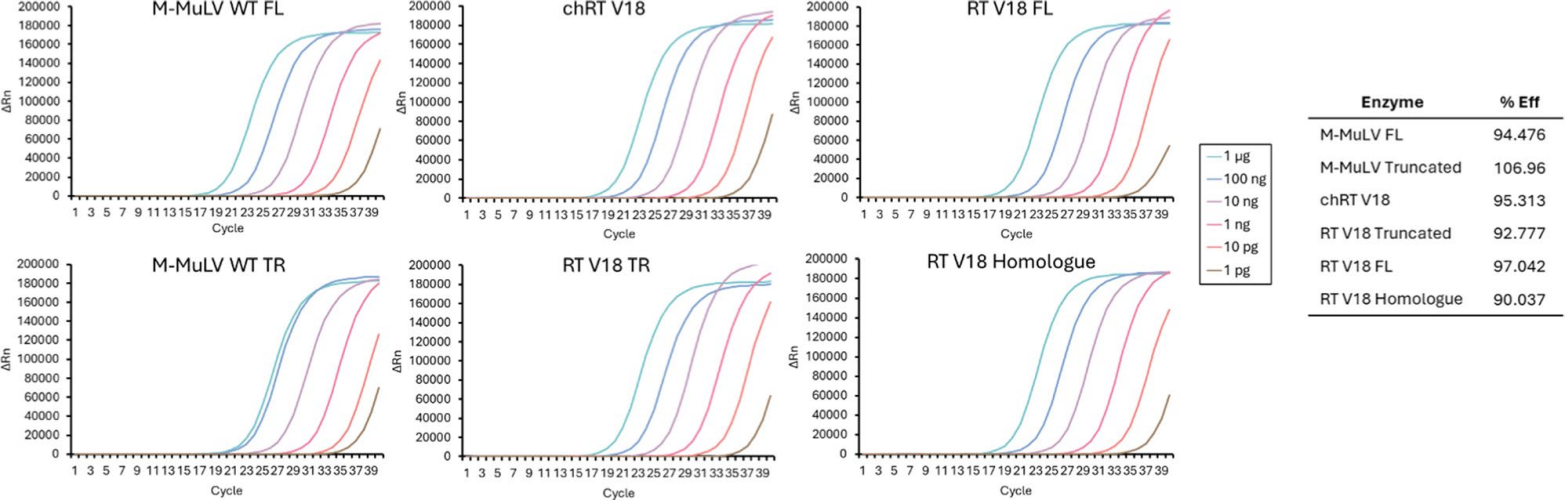
Functional evaluation of chRT V18 and its structural analogs under high-temperature conditions. RT activity was assessed using two-step RT-qPCR, with cDNA synthesis performed at 70 °C for 1 min using mouse liver total RNA primed with oligo(dT). Representative amplification curves are shown for six recombinant enzymes: full-length M-MuLV RT (M-MuLV WT FL), truncated M-MuLV RT lacking the C-terminal domain (M-MuLV WT TR), the chimeric enzyme chRT V18, truncated RT V18 (RT V18 TR), full-length native RT V18 (RT V18 FL), and a closely related RT V18 homologue. Standard curves were generated using five 10-fold RNA dilutions (starting from 1 µg). Performance was evaluated by qPCR amplification of the mouse Rpl27 transcript. Calculated coefficients of determination (R²) and amplification efficiencies are indicated for each enzyme and summarized in Table 1

Enzyme performance was first assessed under stringent conditions using a two-step RT-qPCR assay with reverse transcription performed at 70 °C for 1 min. Under these conditions, all enzymes except M-MuLV WT TR supported efficient cDNA synthesis across the dilution series, exhibiting comparable amplification kinetics and efficiencies (Fig. 6; Table 1). The loss of activity observed for the truncated M-MuLV WT enzyme, particularly at high RNA inputs, contrasted with the sustained performance of RT V18 TR, RT V18 FL, and chRT V18. These findings indicate that while truncation does not inherently preclude high-temperature activity, the sequence context is a critical determinant.

**Fig. 7:**
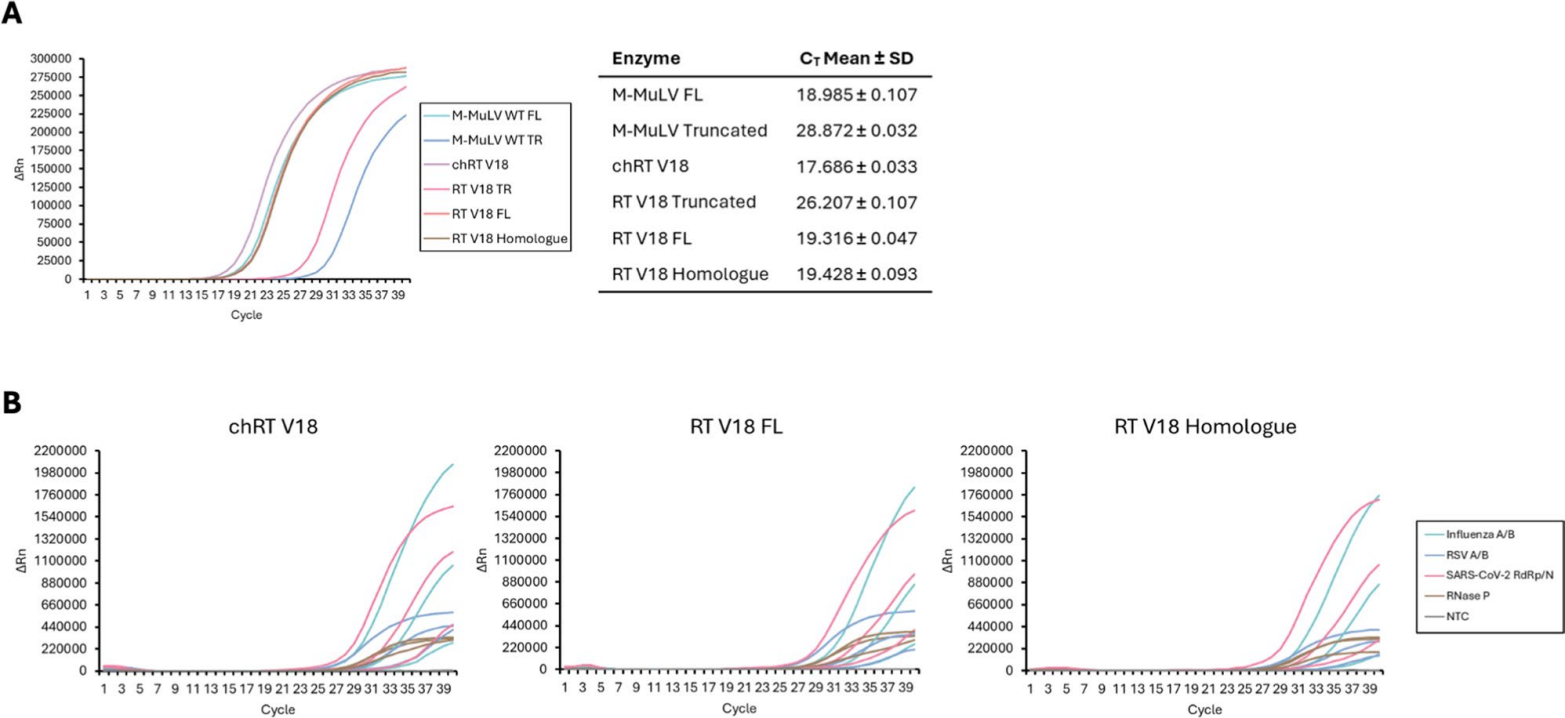
Functional robustness of chRT V18 and its structural analogs under high-stringency conditions and multiplex diagnostic testing. (**A**) RT activity was assessed using two-step RT-qPCR, with cDNA synthesis performed at 70 °C for 1 min using 5 µg of mouse liver total RNA primed with oligo(dT). Amplification curves are shown for six recombinant enzymes: full-length M-MuLV RT (M-MuLV WT FL), truncated M-MuLV RT lacking the C-terminal domain (M-MuLV WT TR), the chimeric enzyme chRT V18, truncated RT V18 (RT V18 TR), full-length native RT V18 (RT V18 FL), and a closely related RT V18 homologue. cDNA synthesis efficiency was quantified by qPCR targeting the mouse Rpl27 transcript. Corresponding C_T_ values are summarized in the table. (**B**) One-step multiplex RT-qPCR performance of chRT V18, RT V18 FL, and the RT V18 homologue was evaluated using a clinically validated IVD assay targeting six viral RNA sequences (SARS-CoV-2 *RdRp* and *N* genes, influenza A, influenza B, RSV A, and RSV B) and a human RNase P internal control. Reactions were performed using a 1-minute reverse transcription step at 60 °C and three RNA input levels (500, 50, and 5 copies per target)

At a higher RNA input of 5 µg, chRT V18 consistently yielded the most robust amplification profiles, whereas truncated enzymes showed reduced tolerance (Fig. 7A). Both native RT V18 FL and its natural homologue maintained efficient cDNA synthesis, albeit with slightly greater variability than the chimeric enzyme. RT V18 TR showed reduced cDNA synthesis capacity at this high RNA load. These results indicate that inclusion of the C-terminal RNase H-containing region in the chRT V18 construct improves performance during short, high-temperature reverse transcription at high RNA input. Finally, chRT V18, RT V18 FL, and RT V18 homologue were evaluated in a clinically relevant context using an IVD-validated multiplex one-step RT-qPCR assay targeting six viral RNA sequences (SARS-CoV-2 RdRp and N, influenza A and B, and RSV A and B), together with an internal human RNase P control, using a 1 min reverse transcription step at 60 °C. All three enzymes consistently detected viral targets across input levels of 500, 50, and 5 RNA copies per target, with stable amplification of the internal control (Fig. 7B). No systematic differences in detection sensitivity were observed between the three enzymes under these conditions. Together, these results indicate that the key diagnostic properties of chRT V18 are largely encoded within the transferred catalytic core, although the reduced tolerance of truncated variants shows that the C-terminal RNase H-containing region contributes materially to robustness under demanding reaction conditions. This supports chimeric RT prospecting as a streamlined and effective approach for identifying high-performance reverse transcriptases with properties directly relevant to molecular diagnostic workflows.

**Table 1:** Comparative performance of RT V18-derived and reference enzymes under stringent RT-qPCR conditions. Efficiencies were calculated from standard curves derived from five 10-fold RNA dilutions. R² values reflect linear regression fit

Enzyme	Format	RT protocol	RNA input (µg)	Mean efficiency (%)	*R*²	Performance summary
M-MuLV WT FL	Full-length	1 min at 70 °C	1 → 10⁻⁵	~ 95	≥ 0.99	Stable
M-MuLV WT TR	Truncated	1 min at 70 °C	1 → 10⁻⁵	—	—	Inefficient at high RNA loads
RT V18 TR	Truncated	1 min at 70 °C	1 → 10⁻⁵	~ 93	≥ 0.99	Stable
RT V18 FL	Full-length	1 min at 70 °C	1 → 10⁻⁵	~ 90	≥ 0.99	Stable
RT V18 homologue	Full-length	1 min at 70 °C	1 → 10⁻⁵	~ 97	≥ 0.99	Stable
chRT V18	Chimeric	1 min at 70 °C	1 → 10⁻⁵	~ 95	≥ 0.99	Most robust

### chRT V18 supports high-temperature RT-LAMP amplification beyond conventional RT limits

Given its inhibitor tolerance, broad temperature range, and rapid cDNA synthesis kinetics, the versatility of chRT V18 in different diagnostic settings was evaluated by assessing its compatibility with high-temperature RT-LAMP. Most RT-LAMP assays rely on strand-displacement DNA polymerases with engineered reverse transcription activity, largely because conventional reverse transcriptases lack sufficient thermostability to operate at typical LAMP temperature ranges (60–65 °C) [41, 42]. In addition, conducting LAMP amplification at higher temperatures (≥ 68 °C) improves assay specificity by suppressing primer-driven non-specific amplification. Still, such conditions are even less compatible with the operating temperature limits of standard reverse transcriptases [43].

**Fig. 8:**
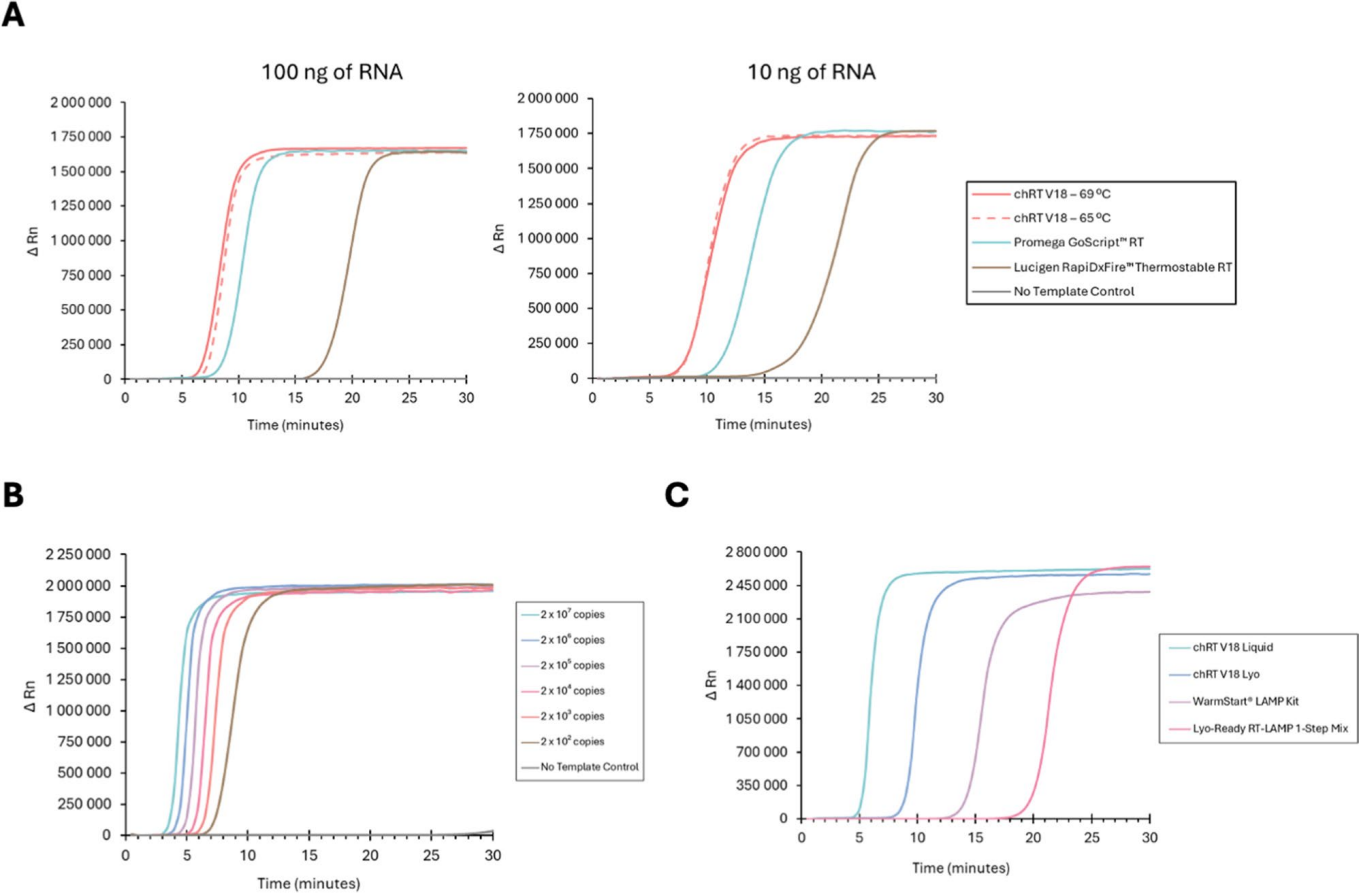
High-temperature RT-LAMP performance of chRT V18. (**A**) Comparison of chRT V18 and two commercial reverse transcriptases in an RT-LAMP assay targeting mouse β-actin mRNA. chRT V18 was evaluated at both 69 °C and 65 °C, whereas the commercial enzymes were evaluated at their manufacturer-recommended temperature of 65 °C. Reactions were performed using 100 ng or 10 ng of mouse total RNA. (**B**) Analytical sensitivity of chRT V18 in high-temperature RT-LAMP. Amplification was evaluated across a dilution series down to approximately 200 target copies. (**C**) Benchmarking of liquid and lyophilized chRT V18-based master mix formulations against two commercial RT-LAMP master mixes under identical reaction conditions

To determine whether chRT V18 could withstand RT-LAMP under these more stringent conditions, the enzyme was benchmarked against two commercially available reverse transcriptases. The results demonstrated that chRT V18 revealed rapid and robust amplification kinetics at both 69 °C and 65 °C. In contrast, the commercial enzymes, evaluated under their manufacturer-recommended conditions at 65 °C, showed delayed RT-LAMP amplification compared with chRT V18 (Fig. 8A). The efficiency and sensitivity of high-temperature RT-LAMP using chRT V18 were evaluated using a dilution series of MS2 genomic RNA, amplified at 69 °C. chRT V18 enabled reliable amplification down to approximately 200 target copies, while no amplification was detected in no-template controls (Fig. 8B). Subsequently, chRT V18 was benchmarked against two commercial RT-LAMP master mixes using both liquid and lyophilized chRT V18-based master mix formulations. Under high-temperature reaction conditions (69 °C), chRT V18 consistently produced an earlier time-to-result and faster reaction kinetics than the commercial alternatives, which followed the manufacturer’s recommended reaction temperature of 65 °C, with only a modest delay observed upon lyophilization and rehydration (Fig. 8C). Together, these results demonstrate that chRT V18 supports efficient and sensitive RT-LAMP amplification at elevated temperatures, which are typically incompatible with conventional reverse transcriptases, thereby extending the operational window of RT-LAMP toward higher specificity and improved analytical robustness.

### Validation of chRT V18-based one-step RT-qPCR master mixes for diagnostic performance

To evaluate whether the biochemical and kinetic advantages of chRT V18 translate into faster and more reliable diagnostic performance, the enzyme was formulated into one-step RT-qPCR master mixes in both liquid and lyophilized formats. These formulations were then assessed against analytically and clinically relevant criteria, with reverse transcription performed at 60 °C for 1 min. The performance of chRT V18 was compared against an IVD-approved reference assay, the NZYtech SARS-CoV-2 One-Step RT-qPCR Kit II, using standardized protocols and clinically relevant sample matrices.

Analytical sensitivity was assessed through limit-of-detection (LoD) analysis using SARS-CoV-2 RNA spiked into nucleic acids extracted from multiple clinically relevant matrices, including blood, nasopharyngeal swabs, urine, and cervical swabs. Each experiment was performed at least in triplicate at different timelines by different operators. Analytical sensitivity was defined as the lowest concentration detected in ≥ 95% of replicates. chRT V18-based master mixes achieved a 100% positive detection rate and consistently achieved a limit of detection of 0.25 copies µL⁻¹ (250 copies mL⁻¹) or five viral RNA copies per reaction. High analytical precision was observed at the LoD (*RdRp*: mean C_T_ = 33.65, SD = 0.53, CV = 1.58%; *N* gene: mean C_T_ = 34.08, SD = 0.61, CV = 1.79%; *n* = 96), across all tested matrices (Table S7). These results indicate that the rapid reverse transcription kinetics and elevated-temperature compatibility of chRT V18 do not compromise analytical sensitivity.

Next, assay precision and robustness were evaluated across independent runs, enzyme batches, operators, instruments, laboratories, and clinical matrices, using both liquid and lyophilized chRT V18 master mix formulations. Across all conditions, 100% detection repeatability was observed, with low intra-assay variability at 3x LoD (CV: 1.30% *RdRp*, 1.79% *N* gene; *n* = 96) and at 30x LoD (CV: 1.14% *RdRp*, 1.80% *N* gene; *n* = 96), and minimal inter-assay variability, all within accepted diagnostic thresholds (Table S8). These results indicate that chRT V18-based master mixes maintain consistent performance under diverse operational conditions, including short reverse transcription steps.

Clinical performance was assessed using a panel of 32 previously characterized SARS-CoV-2 clinical samples (16 positive and 16 negative) spanning a representative range of viral loads. chRT V18-based assays showed full concordance with the IVD-approved reference test, with no observed false-positive or false-negative results (Table S9). C_T_ values were highly correlated between the assays, indicating equivalent clinical sensitivity and specificity, whereas PPA (Positive percent agreement with 95% confidence interval) was 100% for *RdRp* and *N* target genes for SARS-Cov2 virus, and NPA (Negative percent agreement with 95% confidence interval) was 100% for all target SARS-CoV-2 virus. Notably, the chRT V18-based assays achieved equivalent diagnostic performance with a 1-minute reverse transcription step at 60 °C, whereas the IVD-approved reference assay was run according to its standard-validated protocol, which includes a 10-minute reverse transcription step at 50 °C. This demonstrates that chRT V18 enables significant reductions in total assay time without compromising sensitivity, accuracy, or robustness. Together, these data show that chRT V18 supports clinically accurate one-step RT-qPCR while enabling a marked reduction in reverse transcription time, establishing a direct link between enzyme architecture, reaction speed, and diagnostic performance.

## Discussion

Reverse transcriptases used in molecular diagnostics must meet a stringent set of requirements, including sensitivity, tolerance to inhibitory matrices, speed, and compatibility across amplification formats. Here, we show that systematic mining of MuLV RT natural diversity, combined with catalytic-core chimerism, provides a practical route to recover RT phenotypes that integrate several of these traits within a single enzyme framework. Focusing on a structurally and functionally central region of the RT polymerase domain [32, 33], we sampled natural sequence diversity while minimizing redundancy. The resulting variants encompassed both substitutions previously associated with enhanced thermostability or with primer-template interactions, as well as novel sequence combinations without prior functional annotation [6, 7, 22, 34–36]. The broad range of expression yields and solubility observed across the chimeric library underscores the strong influence of sequence context on enzyme stability and heterologous expression [38, 39]. Together, these observations show that catalytic-core chimerism can be used as a practical means to recover diagnostically relevant RT phenotypes from natural sequence diversity while preserving deployable protein expression.

Thermostability screening identified a subset of variants that retain structural integrity at elevated temperatures. Functional assays further revealed that chRT V9, chRT V14, and chRT V18 maintain productive reverse transcription at temperatures up to 70 °C, a range that exceeds that of most RTs currently used in diagnostics [22]. Importantly, thermostability alone did not fully predict enzymatic performance, reinforcing previous observations that reaction context, buffer composition, and enzyme-template interactions strongly influence apparent robustness [20, 33]. Under one-step RT-qPCR conditions, chRT V18 consistently showed the strongest performance among the tested chimeras, particularly at elevated temperatures, suggesting that specific naturally evolved substitutions can preserve catalytic efficiency under thermal stress. Robust inhibitor tolerance further supports the diagnostic relevance of chRT V18. Clinical samples frequently contain substances that impair reverse transcription efficiency [18, 40]. Resistance to inhibition is a critical requirement for reliable testing in minimally processed matrices. chRT V18 displayed inhibitor tolerance comparable to, or exceeding, that of the M-MuLV V1 control for several potent inhibitors, suggesting that its structural features confer resilience to both thermal and chemical stress [12, 13]. A defining feature of chRT V18 is its exceptionally rapid cDNA synthesis kinetics. Efficient reverse transcription was achieved within 1 min across a broad range of RNA inputs and temperatures, without compromising sensitivity or linearity. This property directly addresses a major bottleneck in RT-qPCR workflows, in which reverse transcription is the most time-consuming step. The ability of chRT V18 to combine elevated reaction temperatures with short reverse transcription steps provides a clear route to increasing diagnostic throughput, particularly in high-volume or time-sensitive testing settings.

Functional benchmarking across structural analogs clarified which elements of the chRT V18 architecture drive performance. The diagnostic phenotype was largely preserved in the native RT V18 sequence context, as RT V18 FL and its close homologue, selected from the original 1028-member initial library, remained active at high temperature. However, truncation was not neutral: although RT V18 TR retained activity, its reduced tolerance at high RNA load, together with the loss of function observed for M-MuLV WT TR, indicates that the C-terminal RNase H-containing region contributes materially to robustness under demanding conditions. Notably, transferring the RT V18 catalytic core into the M-MuLV scaffold yielded chRT V18, which exhibited the most resilient performance, consistent with a catalytic core that encodes the key functional profile, while the scaffold architecture enhances operational stability. Collectively, these results support catalytic-core chimerism as a practical approach for capturing naturally evolved RT phenotypes and converting them into high-performance enzymes suitable for a diversity of diagnostic workflows. This was revealed by demonstrating that chRT V18 enabled high-temperature RT-LAMP amplification. Most RT-LAMP implementations rely on strand-displacing DNA polymerases with engineered RT activity, reflecting the limited thermostability of conventional RTs under typical LAMP conditions (60–65 °C) [41, 42]. Operating RT-LAMP at higher temperatures (≥ 68 °C) improves assay specificity by suppressing primer-driven non-specific amplification, but such conditions are generally incompatible with standard RTs. chRT V18 enabled efficient and sensitive RT-LAMP at 69 °C, including in lyophilized format and at low target copy numbers, thereby extending the operational window of RT-LAMP toward higher specificity without added workflow complexity.

Taken together, these findings identify chRT V18 as a high-performance chimeric reverse transcriptase for molecular diagnostics, combining speed, inhibitor tolerance, and broad temperature compatibility across RT-qPCR and RT-LAMP formats. Rather than introducing chimerism as a new engineering concept, this work shows that catalytic-core recombination, when guided by natural sequence diversity and diagnostic performance criteria, can recover diagnostically relevant enzyme phenotypes and translate natural diversity into practical performance gains for next-generation molecular testing workflows. This was clearly demonstrated by showing that the formulation of chRT V18 into one-step RT-qPCR master mixes translates its biochemical advantages directly into analytical and clinical performance. Across multiple clinically relevant matrices, chRT V18-based assays achieved low limits of detection, high precision, and full concordance with an IVD-approved reference test. Notably, equivalent clinical sensitivity and specificity were obtained with a 1-minute reverse transcription step compared with the 10-minute step in the reference assay. This establishes a direct link between enzyme architecture, reaction speed, and diagnostic efficiency.

## Conclusions

This study demonstrates that natural diversity-guided catalytic-core chimerism proved effective for identifying high-performance reverse transcriptases with properties directly relevant to molecular diagnostics. By systematically replacing the functionally critical 405-residue polymerase core of WT M-MuLV RT with naturally evolved homologous sequences, we generated a compact chimeric library that captures broad evolutionary diversity while remaining compatible with established diagnostic workflows. From this approach, three chimeric enzymes, chRT V9, chRT V14, and chRT V18, displayed enhanced performance relative to the parental enzyme across key diagnostic parameters, including expanded temperature tolerance, inhibitor resistance, and efficient cDNA synthesis. Among these, chRT V18 emerged as the most versatile enzyme, maintaining high activity across a wide temperature range, under inhibitor-rich conditions, and during minute-scale reverse transcription without loss of analytical sensitivity.

Functional analyses demonstrated that the diagnostic phenotype of chRT V18 is largely encoded within the transferred catalytic core, although its structural determinants are likely distributed across multiple substitutions within the exchanged 405-residue segment and therefore cannot yet be resolved confidently at the single-residue level. At the same time, incorporation into the M-MuLV backbone further enhances robustness under demanding reaction conditions. Notably, chRT V18 supported both rapid one-step RT-qPCR and high-temperature RT-LAMP, extending reverse transcription into operational regimes that are typically incompatible with conventional RT enzymes. In clinically relevant settings, chRT V18-based master mixes achieved low limits of detection, high precision, and full concordance with IVD-approved assays while enabling substantial reductions in total assay time. Together, these findings establish chRT V18 as a rapid, inhibitor-tolerant, and thermally flexible reverse transcriptase for molecular diagnostics, while showing that catalytic-core chimerism informed by natural sequence diversity can serve as an effective route to recover such multi-trait RT phenotypes.

## Supplementary Information

Below is the link to the electronic supplementary material.

Supplementary Material 1

Supplementary Material 2

Supplementary Material 3

Supplementary Material 4

Supplementary Material 5

Supplementary Material 6

Supplementary Material 7

Supplementary Material 8

Supplementary Material 9

Supplementary Material 10

## Data Availability

All data presented and generated in this work are available upon reasonable request.

## Abbreviations

chRT: Chimeric reverse transcriptase
CVs: Coefficients of variation
cDNA: Complementary DNA
C_T_: Cycle threshold
*E. coli*: *Escherichia coli*
FL: Full length
RNase P: Human ribonuclease P
IMAC: Immobilized metal ion-affinity chromatography
LoD: Limit-of-detection
M-MuLV: Moloney Murine Leukemia Virus
MuLV: Murine Leukemia Virus
NPA: Negative percent agreement
Ppia: Peptidylprolyl isomerase A
RT-qPCR: Quantitative reverse transcription polymerase chain reaction
RT: Reverse transcriptase
RT-LAMP: Reverse transcription loop-mediated isothermal amplification
Rpl27: Ribosomal protein L27
T/P: Template-primer
TR: Truncated
WT: Wild-type
