# Supplementary material for: Natural diversity-guided catalytic-core chimerism engineers a rapid and inhibitor-tolerant reverse transcriptase
Source: J Biol Eng. 2026 May 7;20:109. doi: 10.1186/s13036-026-00692-3 (PMC13317365; doi:10.1186/s13036-026-00692-3)
Supplement: Supplementary file 1 — Supplementary Material 1 [file 13036_2026_692_MOESM1_ESM.docx]

**Natural diversity-guided chimerism yields a rapid and inhibitor-tolerant reverse transcriptase for robust RNA diagnostics**

Inês Fonseca Costa^1, 2, 3^, Vânia Ondina Fernandes^1^, Rita Silva Madureira Simões^1, 2, 3^, Hélvio Simões^1^, Virgínia Maria Rico Pires^1^, Victor Diogo Alves^2, 3^, João Soeiro Teodoro^1^, Pedro Bule^2, 3,*^ and Carlos Mendes Godinho de Andrade Fontes^1, 2, 3^

^1^ NZYtech - Genes & Enzymes, Campus do Lumiar, Building J, 1649-038, Lisbon, Portugal

^2^ CIISA - Centre for Interdisciplinary Research in Animal Health, Faculty of Veterinary Medicine, University of Lisbon, 1300-477, Lisbon, Portugal

^3^ Associate Laboratory for Animal and Veterinary Sciences (AL4AnimalS), 1300-477, Lisbon, Portugal

*****Correspondence: pedrobule@fmv.ulisboa.pt

**Supplementary Material**

**Supplementary Figures**

**
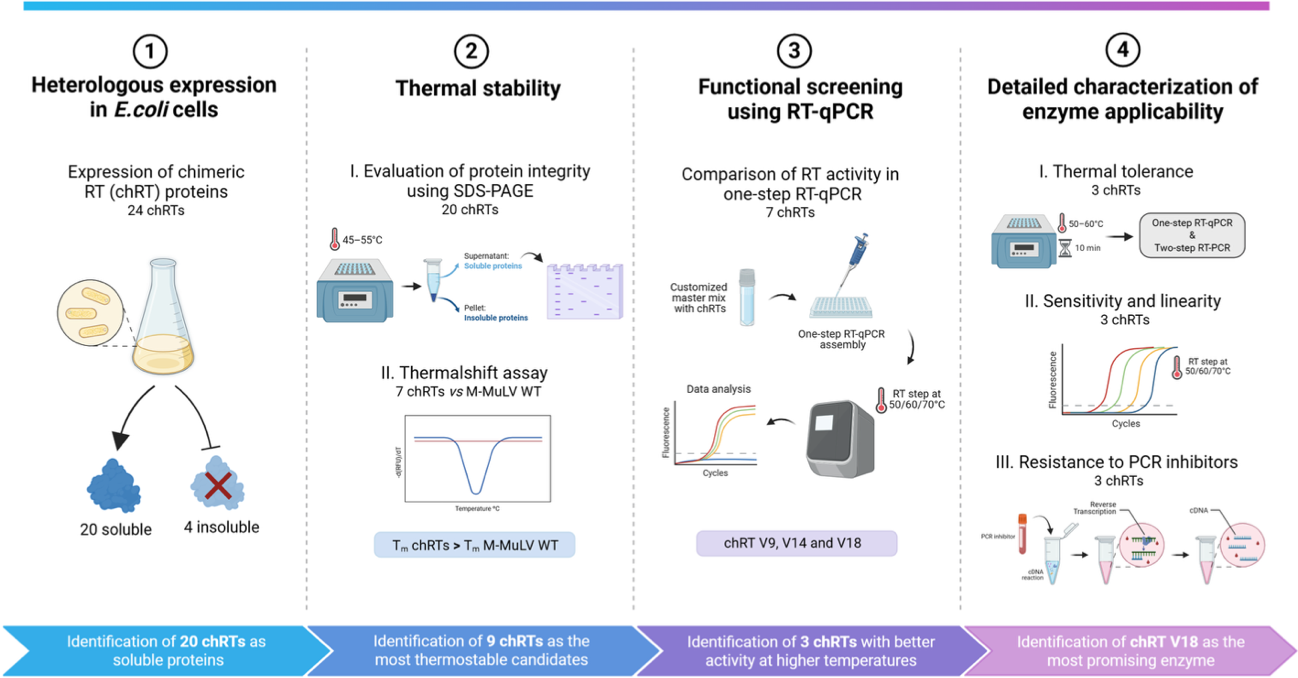
**

**Figure S1. Screening cascade for identification of lead chimeric reverse transcriptases.** From 24 catalytic-core chimeras generated from naturally diverse MuLV-related RTs, 20 yielded soluble recombinant protein and progressed to thermostability screening. Seven variants were then prioritized based on thermal stability and advanced to RT-qPCR-based functional evaluation. Multi-parameter screening identified chRT V9, chRT V14, and chRT V18 as the most robust candidates, with chRT V18 selected as the lead enzyme for downstream validation.


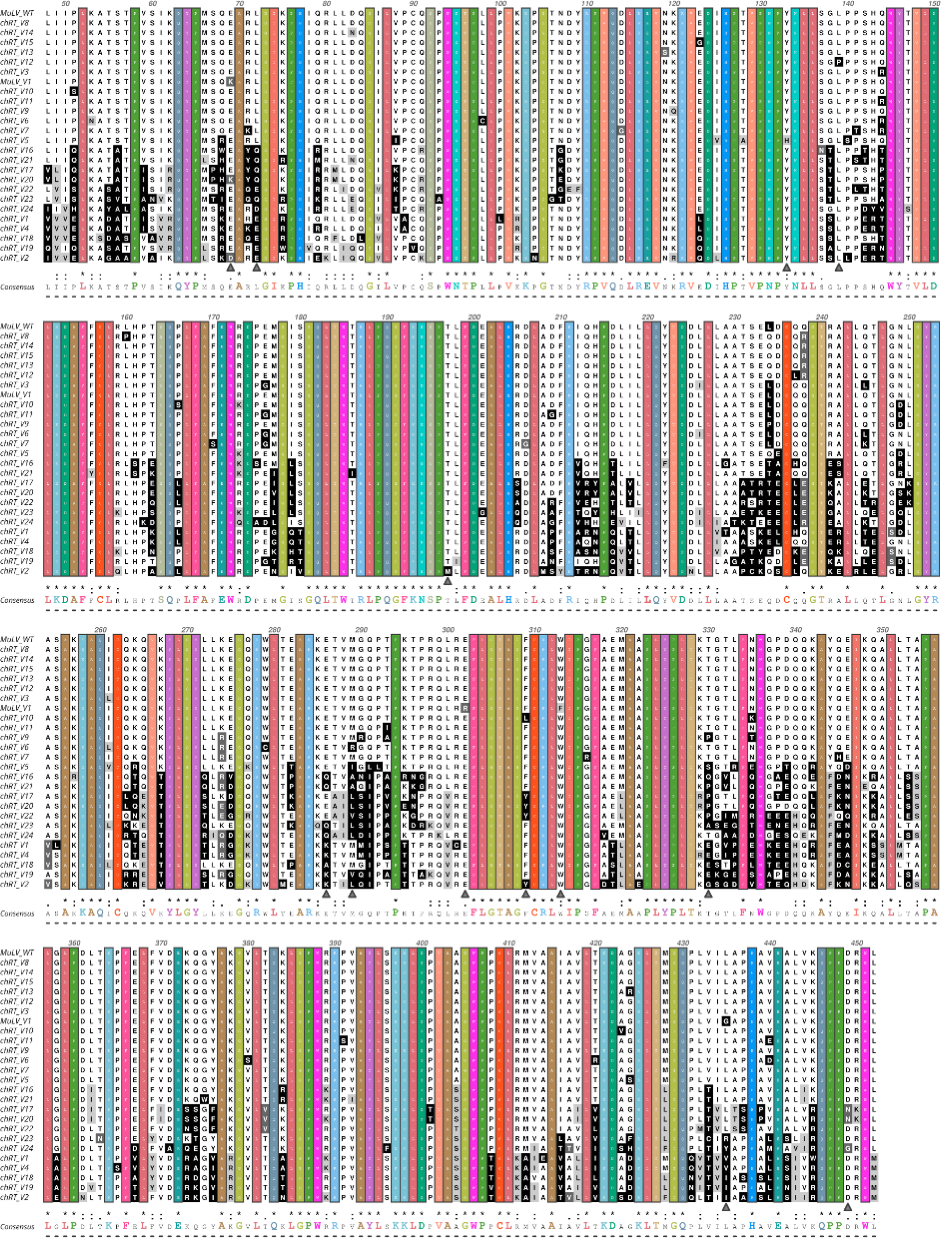


**Figure S2. Multiple sequence alignment of the selected 24 M-MuLV-related variants (chRT V1-V24), M-MuLV WT, and M-MuLV V1.** The multiple sequence alignment of the 405 amino acid regions (Leu48-Leu452) used to produce the chimeric RTs and used RT controls, M-MuLV WT and M-MuLV V1, is shown here. The alignment is displayed using Aline and colored according to the ‘Gecos Flower' coloring scheme. Fully conserved residues are highlighted in their respective colors with white letters, whereas conserved residues are indicated by black letters and highlighted in grey. Both semi-conserved and non-conserved residues are presented with white letters and highlighted in dark grey or black, respectively. The consensus sequence is shown below with colored letters indicating fully conserved residues. Residues previously identified as responsible for improved RT features are marked with black arrows.

**
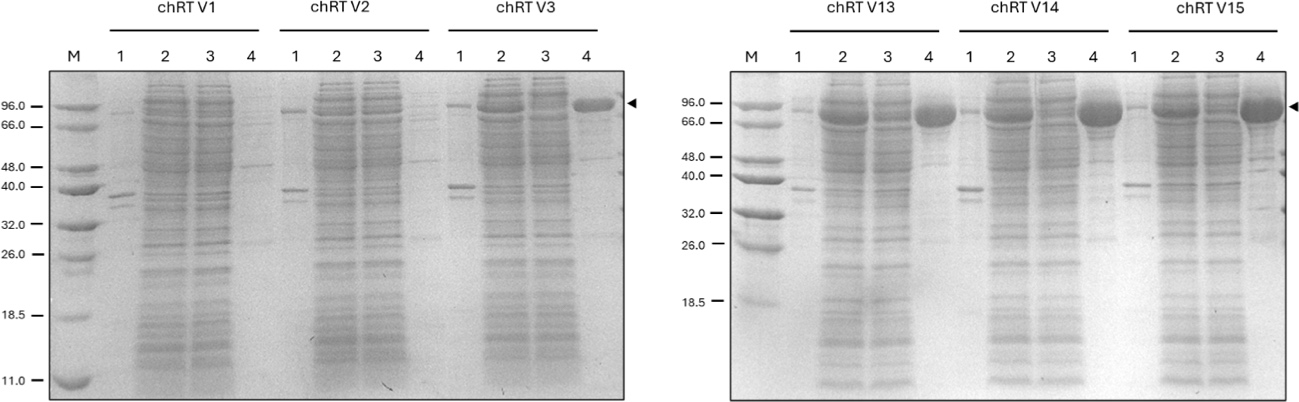
**

**Figure S3. SDS-PAGE analysis of six representative chimeric enzymes (chRT V1-V3, V13-V15) following recombinant expression in *E. coli* and IMAC purification.** For each protein, the gel lanes correspond to: (1) insoluble fraction, (2) soluble fraction, (3) unbound protein collected from the IMAC column, and (4) purified protein after IMAC. M indicates the protein marker. The expected molecular weight (approximately 77 kDa) of the chimeric RTs is highlighted with an arrow. The data illustrate the variability in solubility, integrity, and purification yield among the chimeric enzymes, confirming successful production of functionally relevant enzymes for downstream characterization.


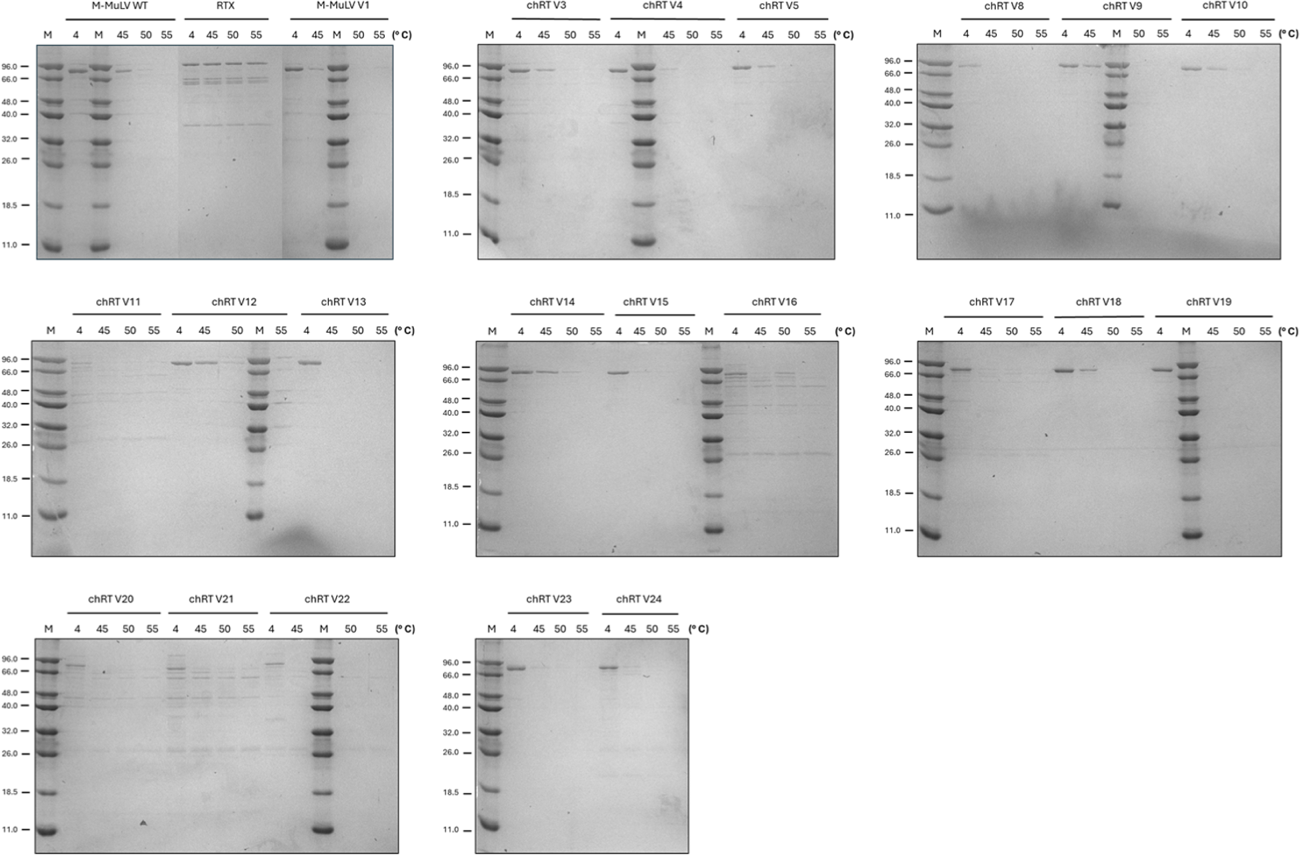


**Figure S4. SDS-PAGE analysis of recombinant RT variants following thermostability screening.** Each variant was incubated for 15 minutes at 4°C, 45°C, 50°C, and 55°C before SDS-PAGE. The protein marker is indicated as M. Some panels were assembled from cropped regions of the same gel image for presentation purposes; these composite boundaries do not affect the qualitative interpretation of protein integrity after heat treatment. The protein thermal stability profile was used to determine candidates for further analysis. This dataset supports the qualitative observations reported in Table S3.


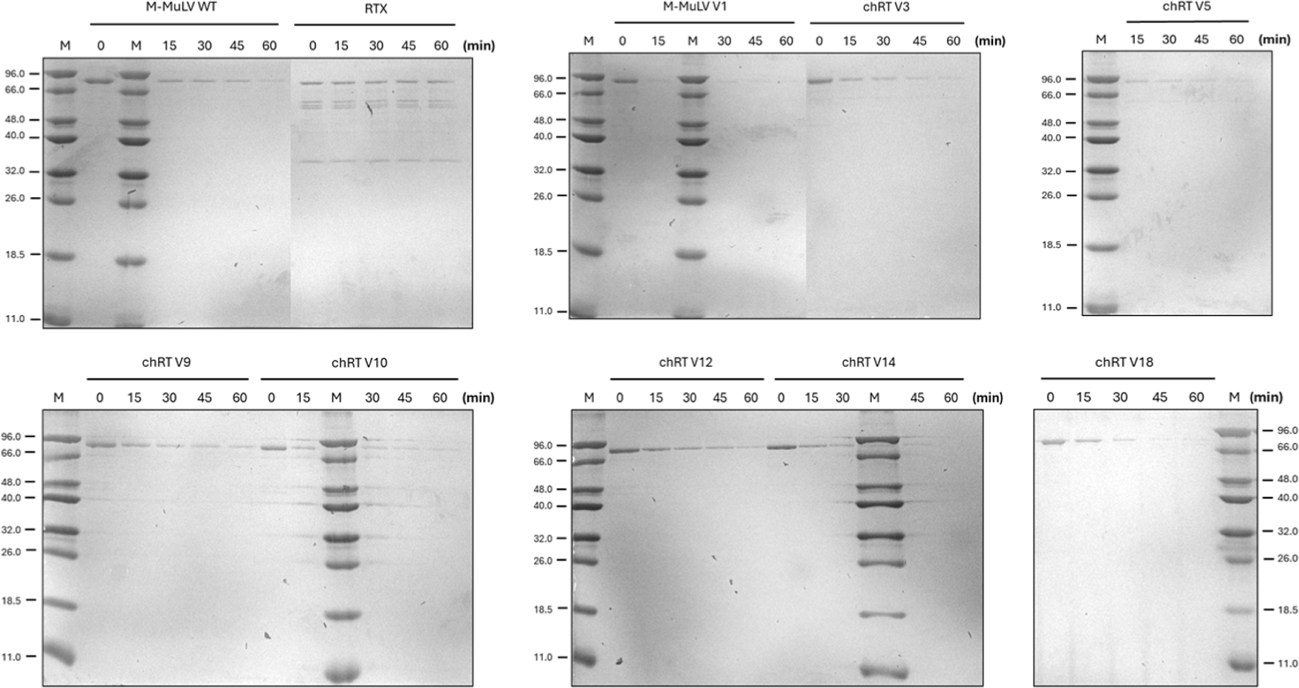


**Figure S5. SDS-PAGE analysis of time-dependent thermal degradation of the nine most thermostable chimeric RT variants.** Gels showing the degradation kinetics of chRT V3-V5, V9, V10, V12, V14, V16, and V18 after incubation at 45°C for 0, 15, 30, 45, and 60 minutes. The protein marker is identified as M. Some panels were assembled from cropped regions of the same gel image for presentation purposes; these composite boundaries are indicated in the figure and do not affect the qualitative interpretation of the degradation patterns. This dataset supports the qualitative observations reported in Table S4.


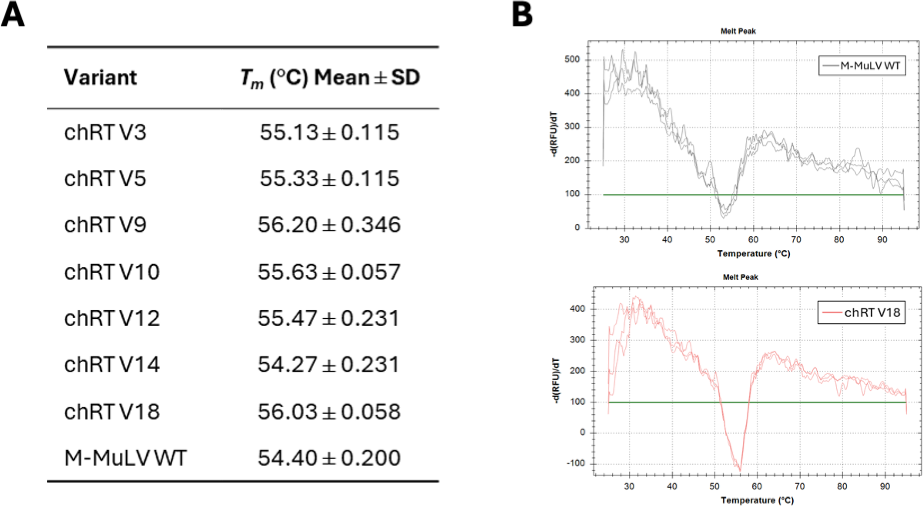


**Figure S6. Protein thermal shift analysis of selected chimeric reverse transcriptases.** Melting temperatures (Tm) of chRT V3, V5, V9, V10, V12, V14, and V18 were determined together with M-MuLV WT RT using the Protein Thermal Shift™ assay. Protein unfolding was monitored by fluorescence during heating from 25°C to 95°C, and T_m_ was defined as the midpoint of the negative melt peak. (A) Data are presented as mean ± SD from three independent technical replicates. (B) Representative melt-peak profiles are shown on the right.

**
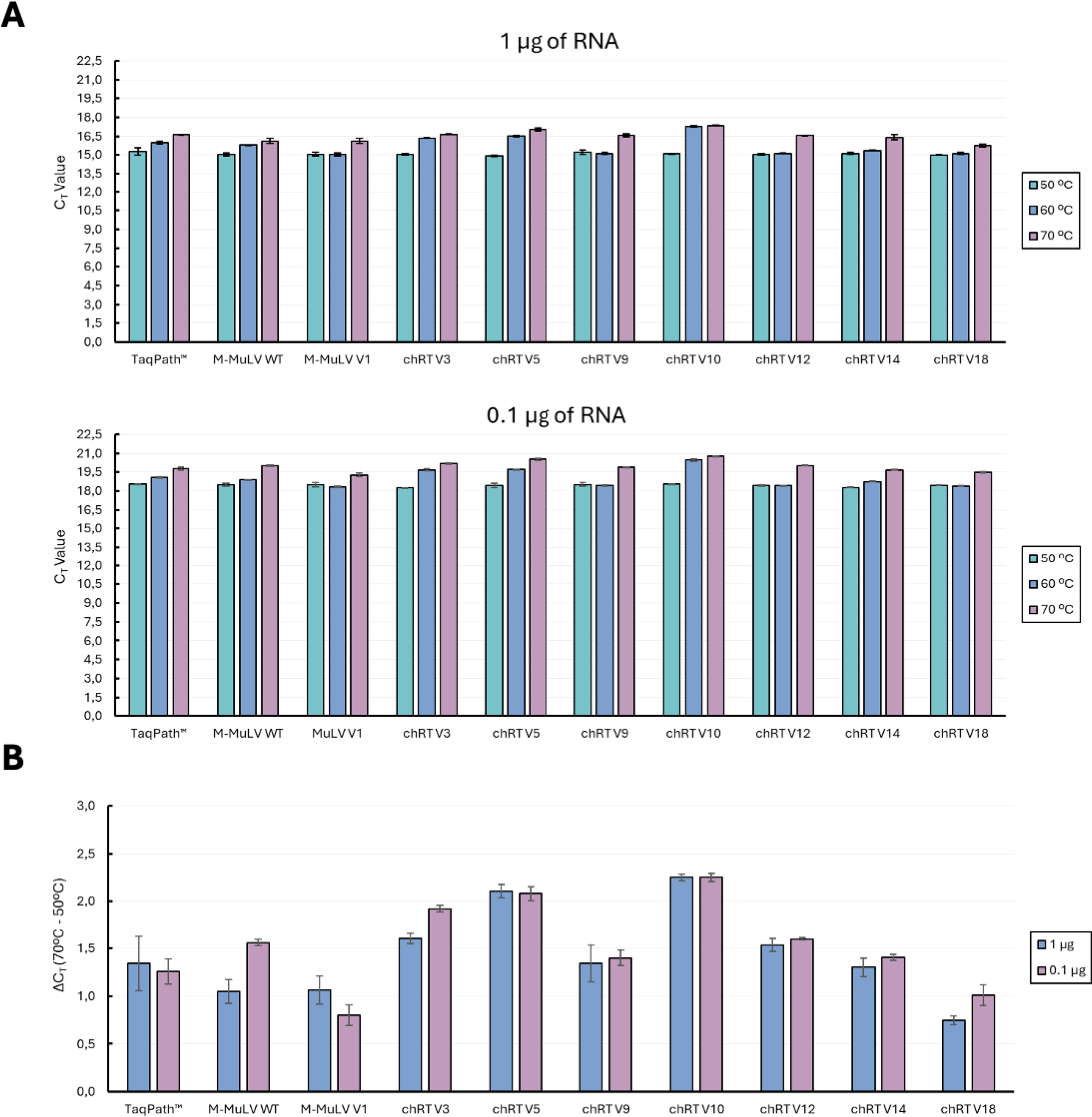
**

**Figure S7. RT-qPCR C_T_ values corresponding to the experiments shown in Figure 2A, illustrating the cDNA synthesis performance of the nine thermostable RT variants and control enzymes at 50°C, 60°C, and 70°C.** Data are presented as mean ± SD from three technical replicates (n = 3). (A) One-step RT-qPCR assays were performed using either 1 µg (top panel) or 0.1 µg (bottom panel) of mouse liver total RNA as template, targeting the mouse *Ppia* housekeeping gene. C_T_ values are shown to allow direct comparison of amplification performance across enzymes and reaction temperatures. (B) ΔC_T_ values (C_T_ at 70°C minus C_T_ at 50°C) calculated from the assays shown in panel A, using either 1 µg or 0.1 µg of mouse liver total RNA as template. Lower ΔC_T_ values indicate greater retention of performance across the tested temperature range.


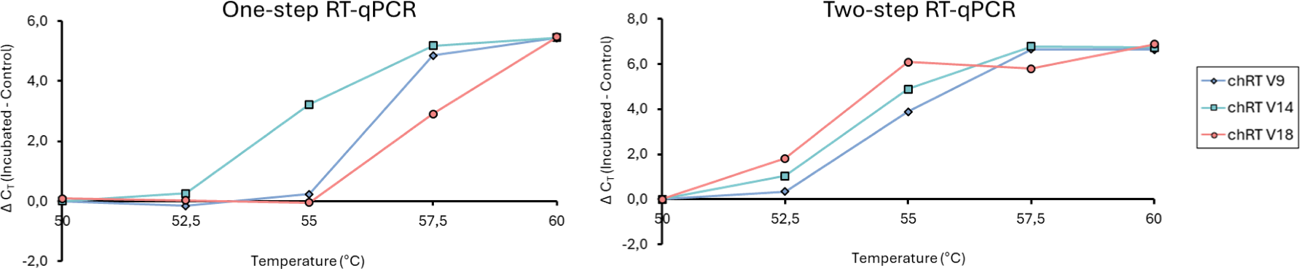


**Figure S8. Thermostability assessment of the best three chimeric RT variants following a 10-minute pre-incubation at temperatures ranging from 50°C to 60°C, with residual activity evaluated by one-step and two-step RT-qPCR.** The three top-performing RT enzymes (chRT V9, V14, and V18) were pre-incubated at 50°C, 52.5°C, 55°C, 57.5°C, and 60°C for 10 minutes before conducting either one-step RT-qPCR (left panel) or two-step RT-qPCR (right panel) assays for mouse *Ppia* detection, using 1 µg of mouse liver total RNA as template. The reverse transcription step in these experiments was performed at 50°C for 10 minutes. A non-incubated control for each enzyme was included. Thermostability is expressed as ΔC_T_, defined as the difference in cycle threshold (C_T_) between the pre-incubated reaction and the non-incubated control; larger ΔC_T_ values indicate greater loss of activity.


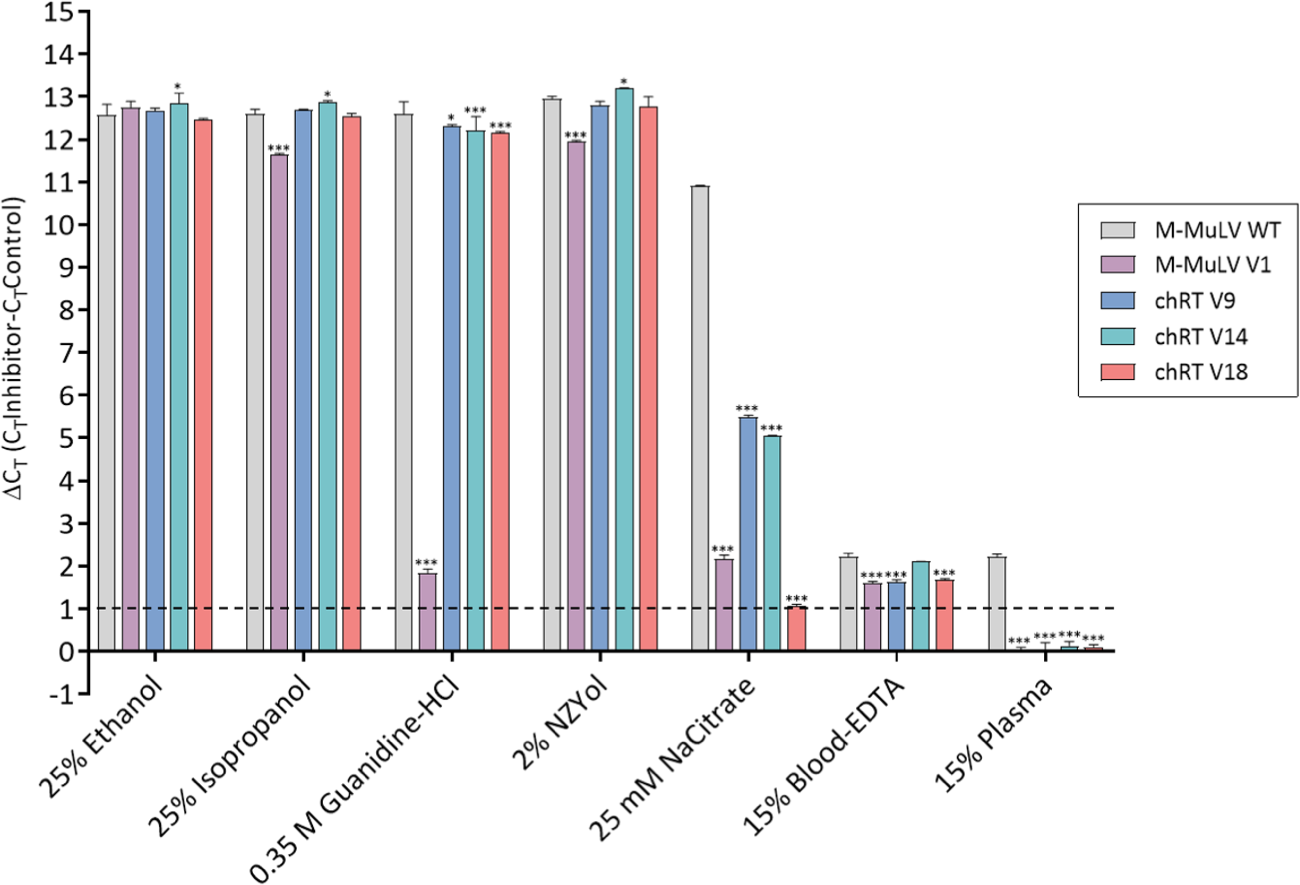


**Figure S9. Inhibitor tolerance of WT, engineered, and chimeric RTs at the alternate inhibitor concentrations relative to Figure 3.** ΔC_T_ values for M-MuLV WT RT, M-MuLV V1, chRT V9, chRT V14, and chRT V18 in the presence of the same clinically relevant PCR inhibitor panel shown in Figure 3. Reverse transcription in the presence of the inhibitors was performed at 50°C for 10 minutes using 1 µg of mouse liver total RNA as template, prior to qPCR detection of cDNA synthesis. Inhibition was quantified as ΔC_T_ (Inhibitor - No inhibitor). The dashed line at ΔC_T_ = 1.0 indicates the threshold for negligible inhibition. For each inhibitor, the concentration shown corresponds to the alternate condition tested relative to Figure 3.

**
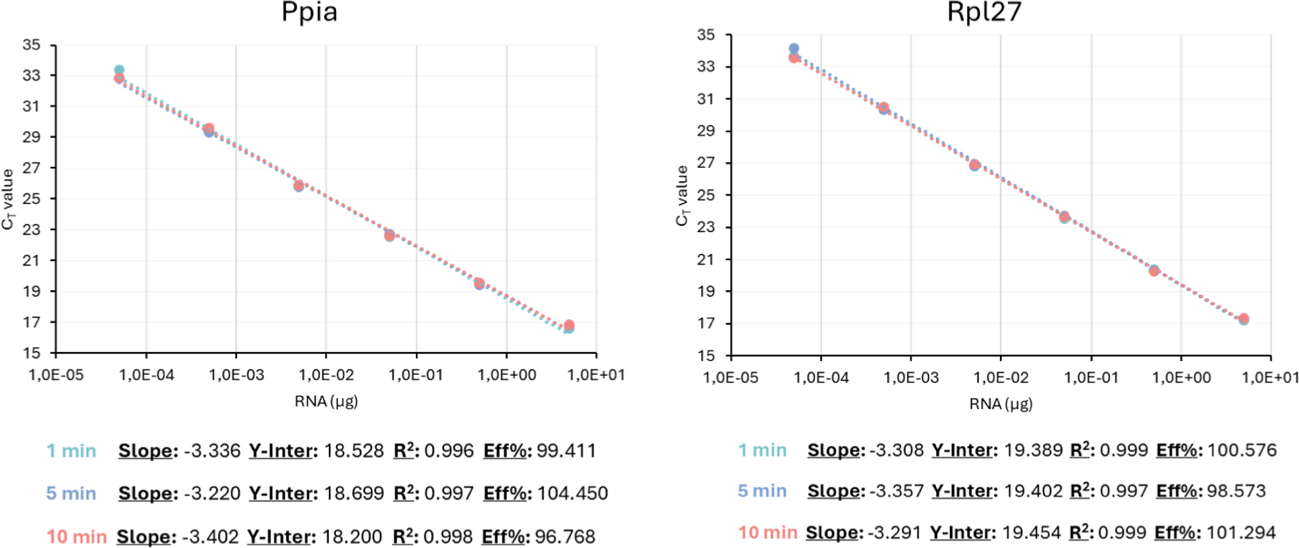
**

**Figure S10. Quantitative analysis of rapid cDNA synthesis by chRT V18 at 60°C.** C_T_-versus-input standard curves corresponding to the datasets shown in Figure 4A. Two-step reverse transcription was performed at 60°C for 1, 5, or 10 minutes using mouse liver total RNA over a six-point 10-fold dilution series, followed by qPCR detection of the mouse Ppia and Rpl27 transcripts. Linear regression parameters, coefficients of determination (R²), and amplification efficiencies are shown for each reverse transcription time.


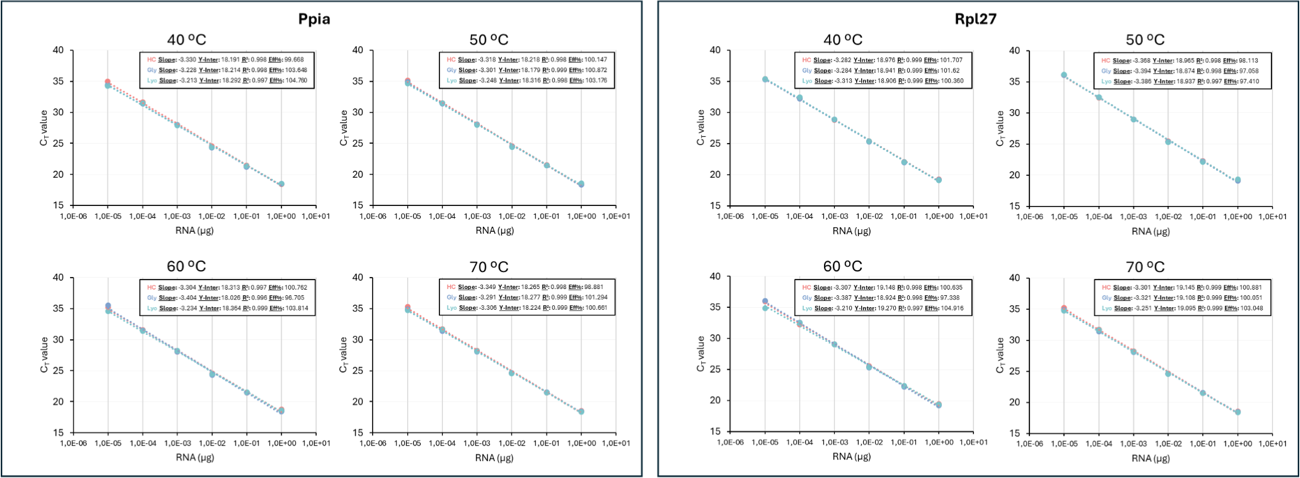


**Figure S11. Quantitative analysis of one-minute cDNA synthesis by chRT V18 across temperature and formulation conditions.** C_T_-versus-input standard curves corresponding to the datasets shown in Figure 5. Two-step reverse transcription was performed for 1 minute at 40°C, 50°C, 60°C, or 70°C using mouse liver total RNA over a six-point dilution series, followed by qPCR detection of the mouse Ppia and Rpl27 transcripts. chRT V18 was tested in three formats: glycerol-free high-concentration (HC), glycerol-preserved standard format (Gly), and lyophilized formulation (Lyo). Linear regression parameters, coefficients of determination (R²), and amplification efficiencies are indicated for each condition.


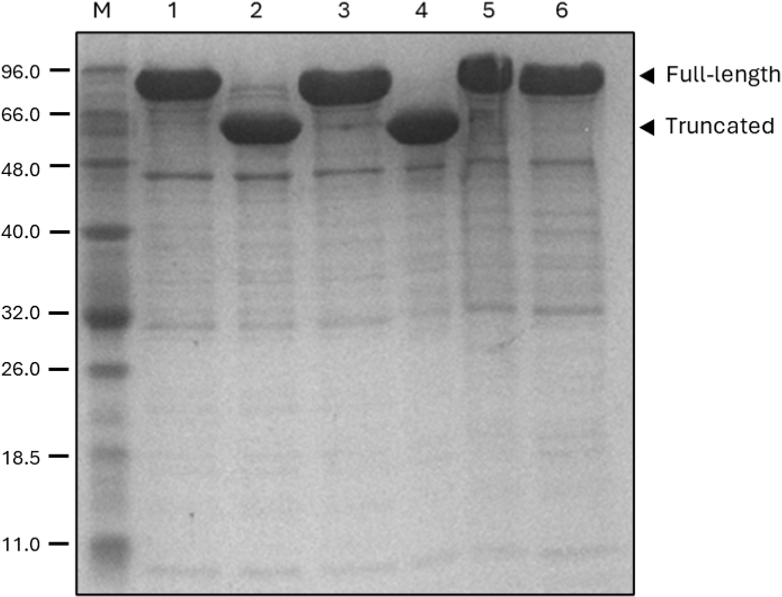


**Figure S12. SDS-PAGE analysis of six RT variants following recombinant expression in *E. coli* and purification.** Each gel lane corresponds to a purified RT variant: (1) M‑MuLV WT RT full-length, (2) truncated M‑MuLV RT, (3) chRT V18, (4) truncated RT V18, (5) RT V18 full-length and (6) RT V18 homologue. M indicates the protein marker. The expected molecular weight of the full-length and truncated RT forms is highlighted with an arrow on the gel.


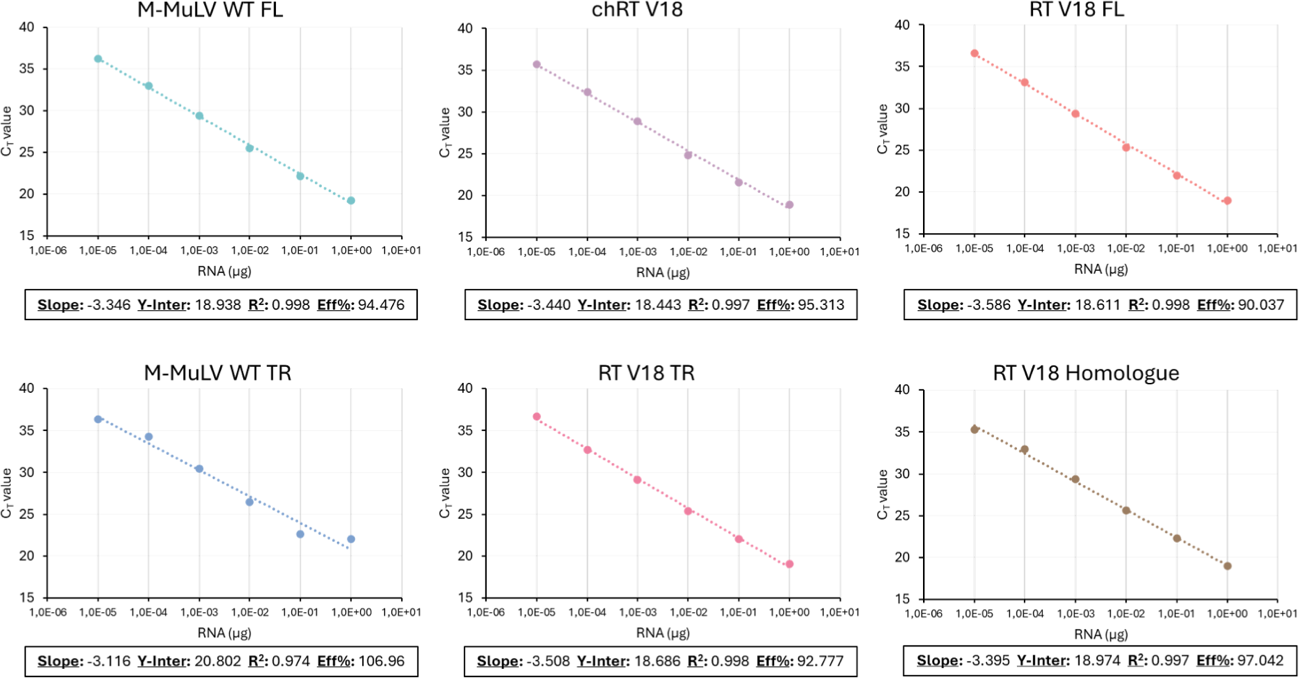


**Figure S13. Quantitative analysis of the functional performance of chRT V18 structural analogs under high-temperature conditions.** C_T_-versus-input standard curves corresponding to the datasets shown in Figure 6. Two-step reverse transcription was performed at 70°C for 1 minute using mouse liver total RNA primed with oligo(dT), followed by qPCR amplification of the mouse Rpl27 transcript. Standard curves are shown for full-length M-MuLV WT RT (M-MuLV WT FL), truncated M-MuLV WT RT (M-MuLV WT TR), chRT V18, RT V18 TR, RT V18 FL, and the RT V18 homologue. Linear regression parameters, coefficients of determination (R²), and amplification efficiencies are indicated for each enzyme.

**Table Legends**

**Table S1. Identification of the selected 24 M-MuLV-related variants.** The table lists the microorganism and the corresponding gene bank accession number for each variant.

**Table S2. Primer and Probe sequences for RT-qPCR and RT-LAMP assays and respective amplicon sizes.**

**Table S3. Summary of predicted molecular weight, extinction coefficient, and protein yield for the 24 chimeric RTs and control enzymes.** The table compiles key biochemical and production parameters for all 24 chimeric RTs (chRT V1-V24), MuLV WT RT, and the engineered variant M-MuLV V1. The theoretical molecular weight (MW, in kDa) predicted molar extinction coefficients at 280 nm (ε, M⁻¹·cm⁻¹), and experimental yields of purified protein (mg per liter of auto-induction culture) are shown.

**Table S4. Thermostability screening of recombinant RT variants.** The 20 successfully expressed RT variants (chRT V1-V24, excluding insoluble or non-expressed ones: chRT V1, V2, V6, and V7) and three control enzymes (M‑MuLV WT RT, M-MuLV V1, and RTX) were incubated for 15 minutes at 4°C, 45°C, 50°C, and 55°C, followed by SDS-PAGE analysis to evaluate protein integrity. Variants chRT V3-V5, V9, V10, V12, V14, V16, and V18 retained structural integrity at 45°C and were selected as the most thermostable candidates (indicated with an asterisk). SDS-PAGE gels used to generate this table are shown in Figure S3.

**Table S5. Time-dependent thermostability assay of the nine most stable RT variants**. The most thermostable RT variants chRT V3-V5, V9, V10, V12, V14, V16, and V18, and three control enzymes (M-MuLV WT RT, M-MuLV V1, and RTX) were incubated at 45°C for 0, 15, 30, 45, and 60 minutes, followed by SDS-PAGE analysis to evaluate enzyme integrity over time. chRT V4 displayed pronounced degradation, whereas chRT V3, V9, and V12 showed stronger stability, maintaining visible bands up to 60 minutes. M-MuLV WT, RTX and M-MuLV V1 were included as controls. SDS-PAGE gels used to generate this table are shown in Figure S4.

**Table S6. Panel of clinically relevant inhibitors and tested concentrations**. This table shows the compounds and respective concentrations used to determine the RTs' resistance to inhibitory reaction conditions. The panel includes residual compounds from the RNA extraction process and compounds commonly present in diagnostic specimens. These concentrations were previously validated to produce stringent (highest) and mild (lowest) inhibitory effects.

**Table S7. Limit of detection of SARS-CoV-2 genomic RNA across different matrices, operators, instruments, and reagent batches.** Experiments were performed in triplicate (n = 3) across independent runs on different days. The limit of detection (LoD) was determined for *RdRp* and *N* genes; mean C_T_, SD, and CV are reported for each condition.

**Table S8. Intra- and inter-assay precision of the chRT V18 master mix.** CVs of C_T_ values for *RdRp* and *N* genes are shown at 3x and 30x LoD, demonstrating 100% detection repeatability and consistent assay performance. Experiments were performed in triplicate (n = 3).

**Table S9. Overall Summary of the Clinical Performance.** Evaluation of the clinical performance of chRT V18-based master mix assays (n = 32), revealing full concordance with the IVD-approved reference test and absence of false-positive or false-negative results.
